# Altitudinal Patterns of Species Diversity and Phylogenetic Diversity across Temperate Mountain Forests of Northern China

**DOI:** 10.1371/journal.pone.0159995

**Published:** 2016-07-25

**Authors:** Wenxin Zhang, Dizhou Huang, Renqing Wang, Jian Liu, Ning Du

**Affiliations:** 1 Institute of Ecology and Biodiversity, School of Life Sciences, Shandong University, Jinan, China; 2 Shandong Provincial Engineering and Technology Research Center for Vegetation Ecology, Shandong University, Jinan, China; 3 Institute of Environmental Research, Shandong University, Jinan, China; Technical University in Zvolen, SLOVAKIA

## Abstract

The spatial patterns of biodiversity and their underlying mechanisms have been an active area of research for a long time. In this study, a total of 63 samples (20m × 30m) were systematically established along elevation gradients on Mount Tai and Mount Lao, China. We explored altitudinal patterns of plant diversity in the two mountain systems. In order to understand the mechanisms driving current diversity patterns, we used phylogenetic approaches to detect the spatial patterns of phylogenetic diversity and phylogenetic structure along two elevation gradients. We found that total species richness had a monotonically decreasing pattern and tree richness had a unimodal pattern along the elevation gradients in the two study areas. However, altitudinal patterns in shrub richness and herbs richness were not consistent on the two mountains. At low elevation, anthropogenic disturbances contributed to the increase of plant diversity, especially for shrubs and herbs in understory layers, which are more sensitive to changes in microenvironment. The phylogenetic structure of plant communities exhibited an inverted hump-shaped pattern along the elevation gradient on Mount Tai, which demonstrates that environmental filtering is the main driver of plant community assembly at high and low elevations and inter-specific competition may be the main driver of plant community assembly in the middle elevations. However, the phylogenetic structure of plant communities did not display a clear pattern on Mount Lao where the climate is milder. Phylogenetic beta diversity and species beta diversity consistently increased with increasing altitudinal divergence in the two study areas. However, the altitudinal patterns of species richness did not completely mirror phylogenetic diversity patterns. Conservation areas should be selected taking into consideration the preservation of high species richness, while maximizing phylogenetic diversity to improve the potential for diversification in the future.

## Introduction

The spatial patterns of species diversity adapted to the main environmental gradient have been a hot issue in ecological and environmental sciences [1]. Comprehending spatial patterns in biodiversity and the causal factors behind them are fundamental to developing sound conservation and management strategies [2, 3], as well as to studying global climate change and predicting its biological impacts on vegetation [4, 5]. In mountain areas, altitude is an important gradient due to large environmental changes across a relatively short geographical range [6]. For example, altitude drives drastic changes in abiotic factors, such as water, temperature, soil properties, and areas [7]. In recent years, altitudinal diversity patterns have been categorized into four major patterns. The most common pattern is a unimodal curve with high richness at intermediate altitudes, which accounts for about 50% of previous studies. Another common pattern is a monotonically decreasing curve with increasing elevation, found in about 25% of previous studies. Monotonically increasing or no obvious trend with increasing elevation accounts for another 25% of previous studies [8]. The variation in species diversity patterns can be caused by many factors, such as climate, productivity, anthropogenic influences, evolutionary history, and biotic interactions, e.g. competition and facilitation [9, 10]. Although there are many proposed hypotheses to explain the altitudinal patterns, the underlying mechanisms remain poorly understood [11].

Beta diversity is important for understanding spatial patterns of species diversity. It can provide insights into the mechanisms driving community assembly and can guide the selection of conservation areas [12, 13]. Beta diversity is used to describe turnover in species composition across spatial and temporal scales [14]. Many methods for calculating beta diversity have been developed [15]. Distance-decay is the slope of the relationship between the similarity in species composition between two communities and the spatial distance separating them [16]. This method is favored for exploring ecological mechanisms because it is sensitive to key spatial processes, such as dispersal limitation [17, 18]. The similarity in species composition is reduced with increased spatial distance [16]. Different traits among plant taxa, such as body size, dispersal ability, and niche width, may have significant effects on their response to environmental gradients [16, 19, 20]. The slope of the relationship between similarity and distance is steeper for plant taxa with weaker dispersal ability and narrow niches [16, 21].

In contrast to the long-term studies on the spatial patterns of species diversity, research on spatial patterns of phylogenetic diversity is still in its infancy [22]. As the availability of phylogenetic information has increased in recent years, ecologists have attempted to apply phylogenetic approaches to traditional diversity analyses [23–25]. Because considerable phylogenetic conservatism has been found in the niche positions of plants, species that have close phylogenetic relationships usually have similar ecological and functional characteristics [26]. If communities are structured mainly by environmental filtering, environments could select species that have certain physiological tolerances, resulting in co-occurring species having close phylogenetic relationships. In contrast, if communities are structured mainly by interspecific competition, species occupying similar ecological niches compete more strongly with one another and cannot co-exist readily, which causes species occurring in a local community to be overdispersed in the phylogeny [23, 27]. Therefore, studying phylogenetic diversity and structure can reveal ecological and evolutionary processes regulating community assembly [23, 28] and provide novel insights into exploring the mechanisms that are driving current diversity patterns [24].

In order to understand altitudinal patterns of species diversity and its potential causes, we used multifaceted methods to examine altitudinal patterns in two temperate mountain systems of northern China. First, we examined patterns of species richness and composition turnover along the altitudinal gradient. Then, we employed phylogenetic approaches to explore altitudinal patterns of phylogenetic diversity and phylogenetic turnover, and compared whether phylogenetic diversity and species diversity patterns are consistent. Furthermore, we examined phylogenetic structure along two elevation gradients to understand the mechanisms that are driving current diversity patterns.

## Materials and Methods

### Study areas

This study was conducted on Mount Tai (N36° 5′–N36° 15′, E117° 5′–E117° 24′) and Mount Lao (N36°5′–N36°19′, E120°24′–E120°42′), which are located in the western and eastern regions, respectively, of Shandong Province of northern China. Mount Tai is one of China's five most famous mountains, and was listed as a UNESCO World Cultural and Natural Heritage site in 1987. The altitude of the main peak is 1532.5 m, which is the highest in Shandong Province. Mount Lao is surrounded by the sea on two sides. It is the highest peak on the Chinese coastline at 1132.7 m. Mount Tai and Mount Lao are both nature reserves in China and play an important role in local development, such as contributing to the tourist industry and providing ecosystem services.

Mount Tai features a warm-temperate continental monsoon climate. On the top of the mountain, annual mean temperature is 5.3°C and annual mean precipitation is 1124.6 mm. At the bottom of the mountain, annual mean temperature is 12.8°C and annual mean precipitation is 715.0 mm. The average frost period lasts 159 d, and the extreme minimum temperature is -27.5°C.The annual relative humidity is 63%. The climate of Mount Lao has a temperate maritime climate owing to the proximity of the ocean. Mean annual precipitation increases with elevation from 726.6 mm to 2103.8 mm. The annual relative humidity reaches 73%. The mean annual temperature is 11.9°C. The average frost period is over 186 d and the extreme minimum temperature is -21.2°C. Mount Tai and Mount Lao are rich in vegetation with temperate deciduous broad-leaved forests and temperate coniferous forests.

### Field sampling

Vegetation studies were carried out on Mount Tai and Mount Lao in August and September 2012 and 2013, with the permission of the scenic spot management committee of Mount Tai and Mount Lao. In order to systematically sample, the altitudinal gradient was divided into 100 m-wide elevation belts. Two or three study plots (20m × 30m) were set up in each elevation belt. The sampling sites were chosen selectively to include mature forests which were representative of typical vegetation types in each altitudinal belt and to be away from slope crests and clough, large rocky outcrops, and stream gullies. A total of 63 plots were sampled on Mount Tai and Mount Lao. All trees in each plot were identified to species and their diameter at breast height (DBH) was recorded if DBH≥5 cm. All shrubs and herbs appearing in each plot also were also identified to species. Each plot was located by using GPS (Garmin 621SC, Garmin Corp). Elevation, slope, aspect, and human disturbance for each plot were also recorded. Total basal area of trees per plot was calculated to indicate canopy density. The assessment of human disturbance was based on the distance from plots to surrounding roads, hiking trails, and infrastructure, the extent of trampling by tourists, and the amount of trash. The intensity of human disturbances was classified into four grades: 1 = no obvious disturbance, 2 = weak disturbance, 3 = medium disturbance, 4 = heavy disturbance.

### Data analysis

In this study, species diversity was defined as the total number of species recorded in each plot. We calculated total richness, tree richness, shrub richness, and herb richness. Beta diversity was determined by the similarity of species composition between two plots and calculated using the Jaccard similarity index [29]:

$$\beta_{j}=(b+c)/(a+b+c)$$

where a is the numbers of species occurring in both sampling units, and b and c are the number of species unique to one sampling unit and to the other sampling unit, respectively. The higher the Jaccard similarity index, the larger the difference in species composition between two plots. β_j_ was calculated separately for trees, shrubs, herbs, and total species.

We used the Qian and Jin‘s (2015) PhytoPhylo megaphylogeny as a backbone to construct phylogenies of angiosperm plants for Mount Tai and Mount Lao, respectively (S1 and S2 Figs). We applied S.PhyloMaker package of R software to generate phylogenies based on Scenarios 3 [30]. Phylogenetic diversity was quantified using Faith’s index, which is defined as total branch length among all taxa in a plot [31]. Phylogenetic beta diversity was measured by using the nearest taxon method [32]. For each species in one sampling unit, the nearest phylogenetic neighbor in another sampling unit was found, and the mean of each sampling unit was calculated. The mean nearest phylogenetic distance between each pair of plots within each mountain was calculated [23, 33].

We measured the community phylogenetic structure using Net Relatedness Index (NRI) [23]. First, mean phylogenetic distance (MPD) between all possible pairs of taxa in each plot was calculated, and then MPD was compared to randomly generated null communities (MPDnull). Finally, standardization by the standard deviation of phylogenetic distances in the null communities was performed. The formula for NRI is given by:

$$NRI=-1\times \frac{\left(MPDsample-MPDnull\right)}{sd(MPDnull)}$$

Positive values of NRI show that co-existing species in a plot are more closely related phylogenetically than expected by chance (phylogenetic clustering). Negative values of NRI show that species appearing in a plot are less phylogenetically related than expected by chance (phylogenetic overdispersion) [33].

In order to detect how species diversity changes along the elevation gradient, linear and quadratic regression analyses were performed. Species richness or phylogenetic diversity was used as the response variable, while altitude was used as the explanatory variable. Distance-decay was used to indicate the spatial patterns of beta diversity [16]. We calculated Jaccard’s similarity index and phylogenetic beta diversity between each pair of plots within each mountain, and then regressed them on altitudinal divergence. In order to identify the main environmental factors affecting plant diversity, Pearson correlation analysis was performed to assess the relationship between alpha diversity (species richness and phylogenetic diversity) and environmental variables, with the altitude, slope, aspect, disturbance, and total basal area as explanatory factors.

Phylogenetic diversity were calculated using Phylocom 4.2 [33]. Regression and correlation analyses were conducted using R Software, version 3.2.2 [34].

## Results

### Altitudinal patterns of species richness and phylogenetic diversity

**Fig 1:**
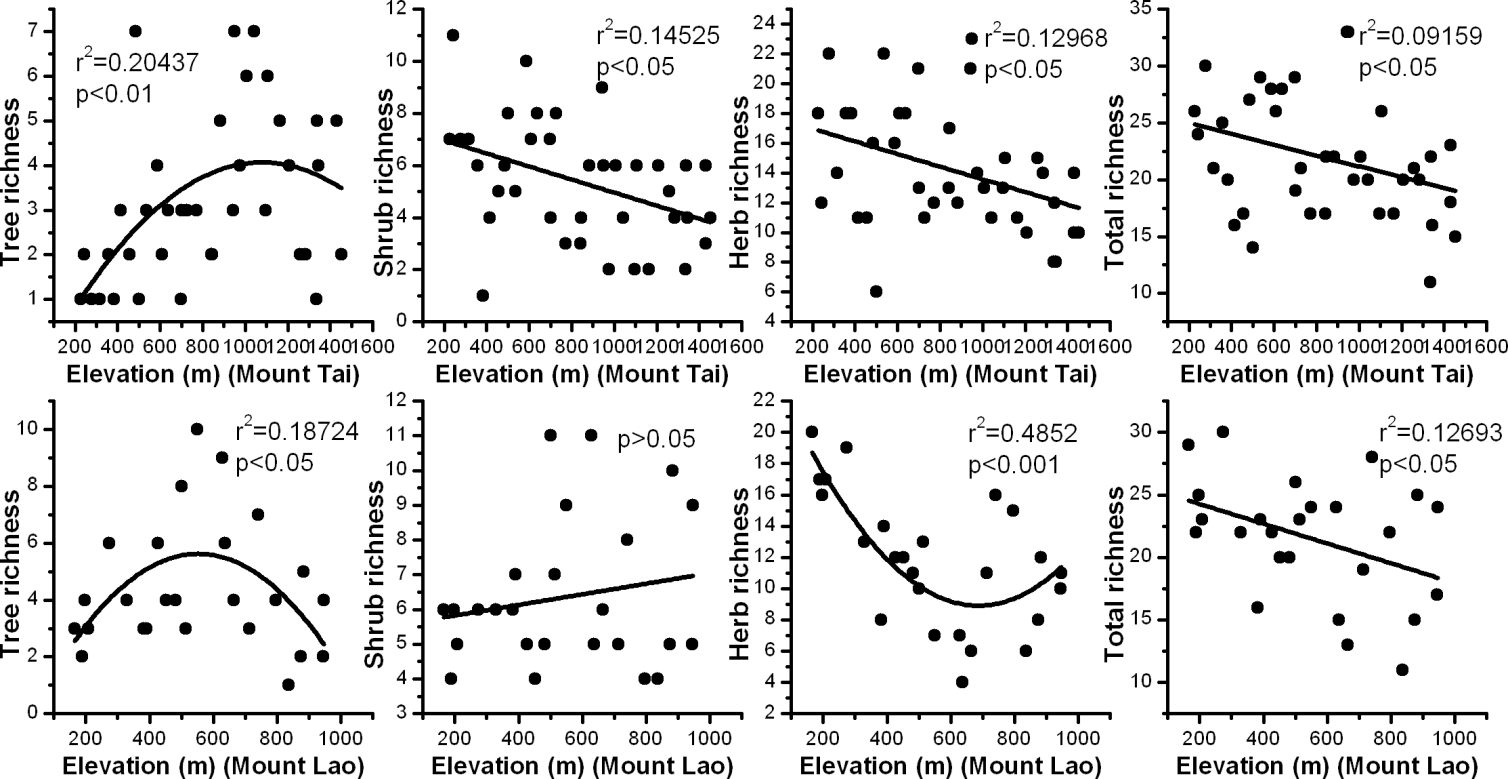
Variation in species richness along elevation gradients for trees, shrubs, herbs, and total species on Mount Tai and Mount Lao, China.

The total species richness on Mount Tai and Mount Lao has a consistent altitudinal pattern. With increasing elevation, the total number of species recorded in each plot decreased linearly. A unimodal pattern was detected for trees in both mountain systems. Trees richness increased and then decreased along altitudinal gradients. Maximum species richness occurred at about 1000 m on Mount Tai and 600m on Mount Lao. Shrubs have a monotonic decline from low to high elevation on Mount Tai. However, we did not found clear patterns for shrubs on Mount Lao. Herb richness exhibited a monotonically decreasing pattern on Mount Tai and inverted hump-shaped pattern on Mount Lao (Fig 1).

**Fig 2:**
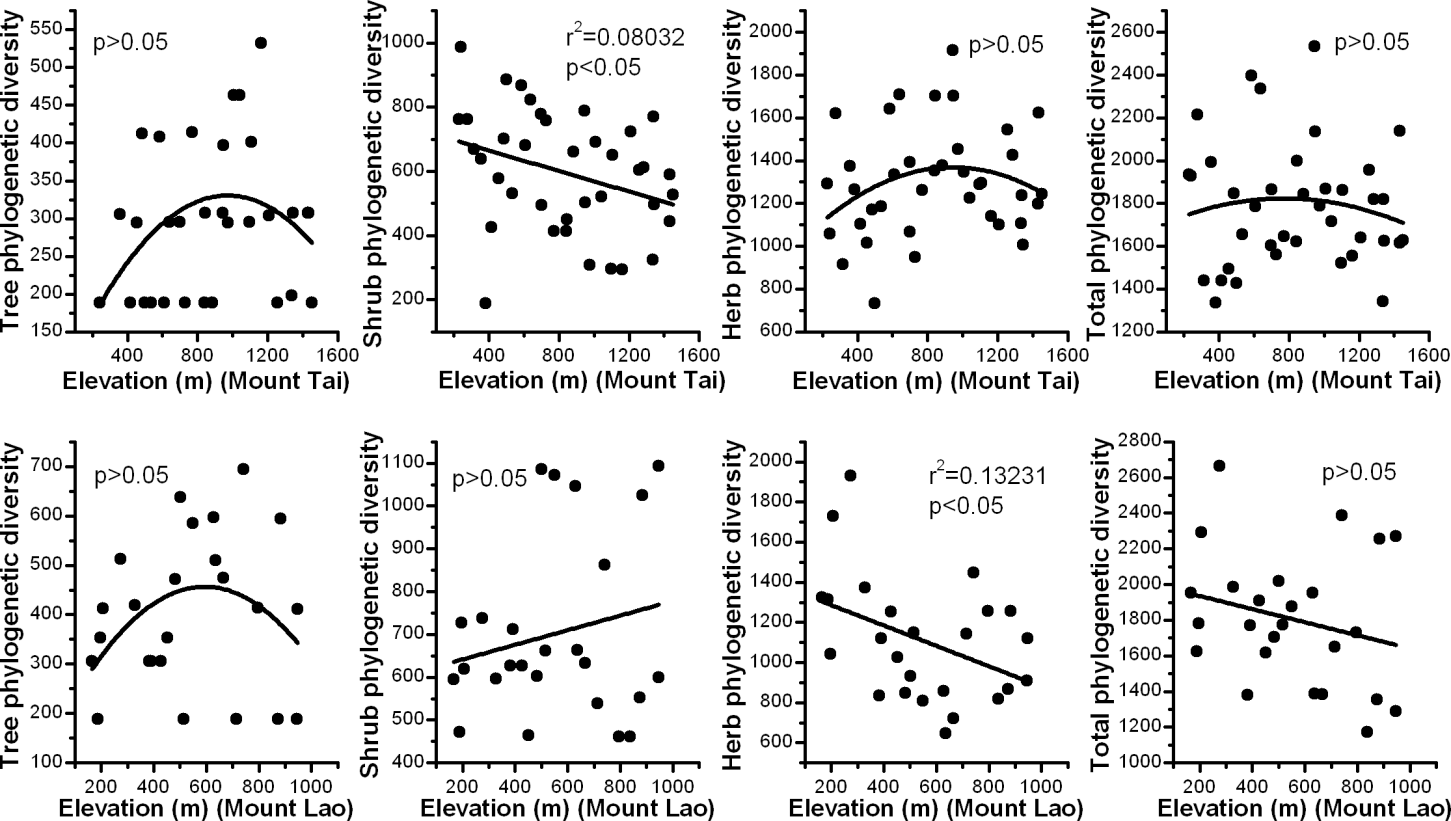
Variation in phylogenetic diversity along elevation gradients for trees, shrubs, herbs, and total species on Mount Tai and Mount Lao, China.

There was no clear trend for total phylogenetic diversity along the elevation gradient in the two mountain systems. For tree species, we also did not found clear patterns along the elevation gradient. A significant negative relationship between the phylogenetic diversity of shrubs and elevation was detected for species on Mount Tai; however, no obvious relationship was detected for species on Mount Lao. For herb species, the monotonically decreasing pattern was only found for species on Mount Lao; no clear pattern was found for species on Mount Tai (Fig 2).

Pearson correlation analysis showed that elevation, human disturbance, and total basal area play an important role in regulating species richness and phylogenetic diversity. Moreover, human disturbance and total basal area had greater impacts on shrubs and herbs than on trees (Table 1).

**Table 1:** Pearson correlation coefficients between alpha diversity and environmental factors.

Diversity type	Elevation	Aspect	Slope	Disturbance	Total basal area
**Mount Tai**					
Total richness	-0.341*	0.071	-0.065	0.364*	-0.354*
Tree richness	0.407*	-0.040	0.156	-0.271	0.280
Shrub richness	-0.410*	-0.014	-0.042	0.392*	-0.263
Herb richness	-0.391*	0.076	-0.104	0.362*	-0.413**
Total phylogenetic diversity	-0.047	-0.155	-0.119	0.320	0.068
Tree phylogenetic diversity	0.161	-0.113	0.070	0.020	0.054
Shrub phylogenetic diversity	-0.324*	0.022	-0.007	0.331*	-0.194
Herb phylogenetic diversity	0.135	-0.164	-0.103	0.177	0.147
**Mount Lao**					
Total richness	-0.399*	0.487*	-0.096	0.376	-0.701**
Tree richness	0.952	0.066	0.758	-0.276	-0.186
Shrub richness	0.178	0.055	0.122	-0.004	-0.394
Herb richness	-0.560**	0.390	-0.171	0.601**	-0.575**
Total phylogenetic diversity	-0.244	0.377	-0.045	0.227**	-0.577
Tree phylogenetic diversity	0.117	0.337	0.176	-0.378	0.006
Shrub phylogenetic diversity	0.205	0.004	0.189	-0.057	-0.339**
Herb phylogenetic diversity	-0.410*	0.326	-0.251	0.485	-0.479**

Asterisks indicate significant effects

*p < 0.05

**p < 0.01

### Altitudinal patterns of species turnover and phylogenetic distance

**Fig 3:**
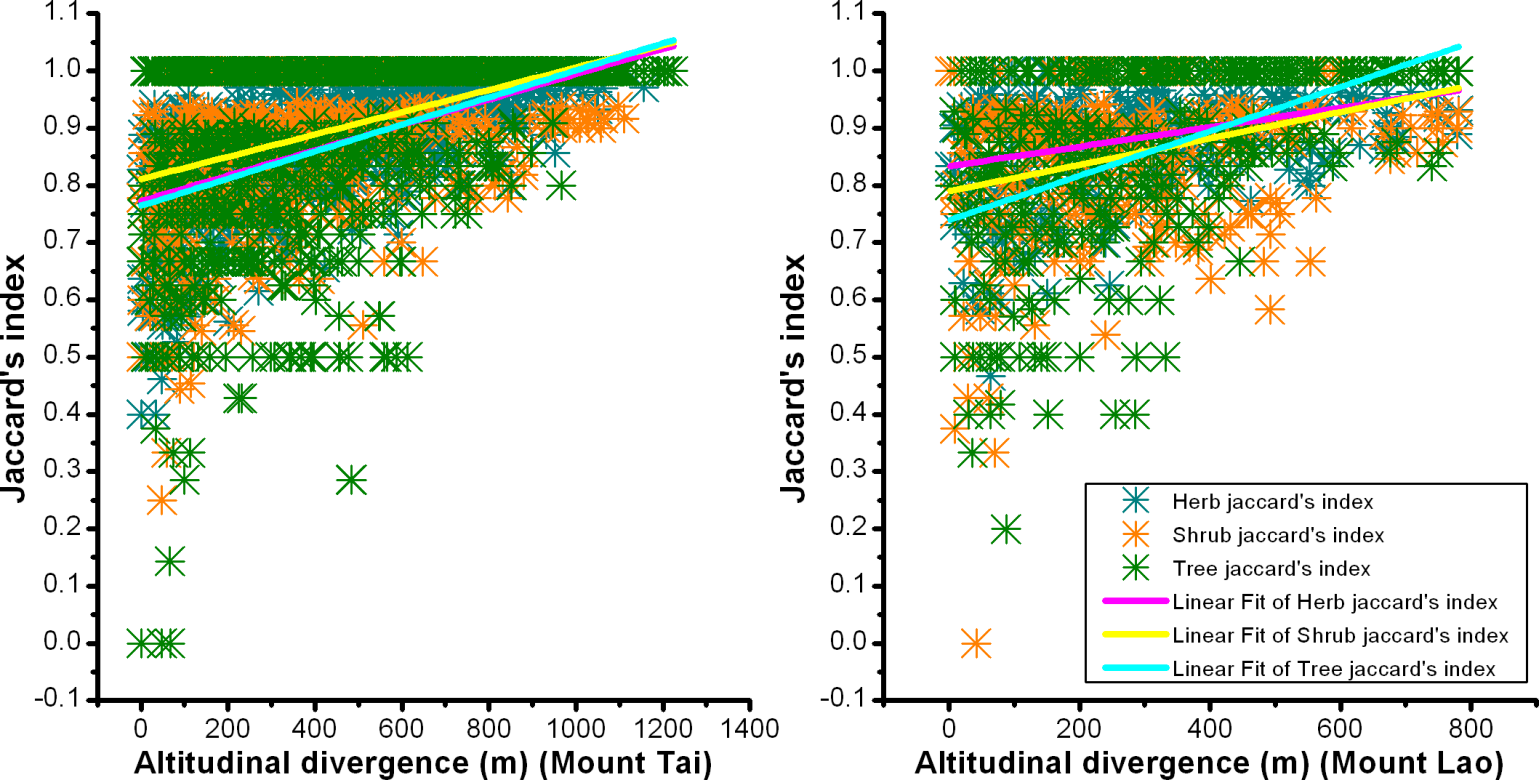
Relationship between Jaccard similarity index and altitudinal divergence for trees, shrubs, and herbs on Mount Tai and Mount Lao, China.

The similarity in species composition between two plant communities decreased as altitudinal divergence increased. The regression slope between Jaccard similarity index and altitudinal divergence was not uniform among different plant groups. On Mount Tai, the regression slope of trees, shrubs, and herbs was 0.000237, 0.000195, and 0.000221, respectively. On Mount Lao, the regression slope of trees, shrubs, and herbs was 0.000388, 0.000231, and 0.000170, respectively (Fig 3).

**Fig 4:**
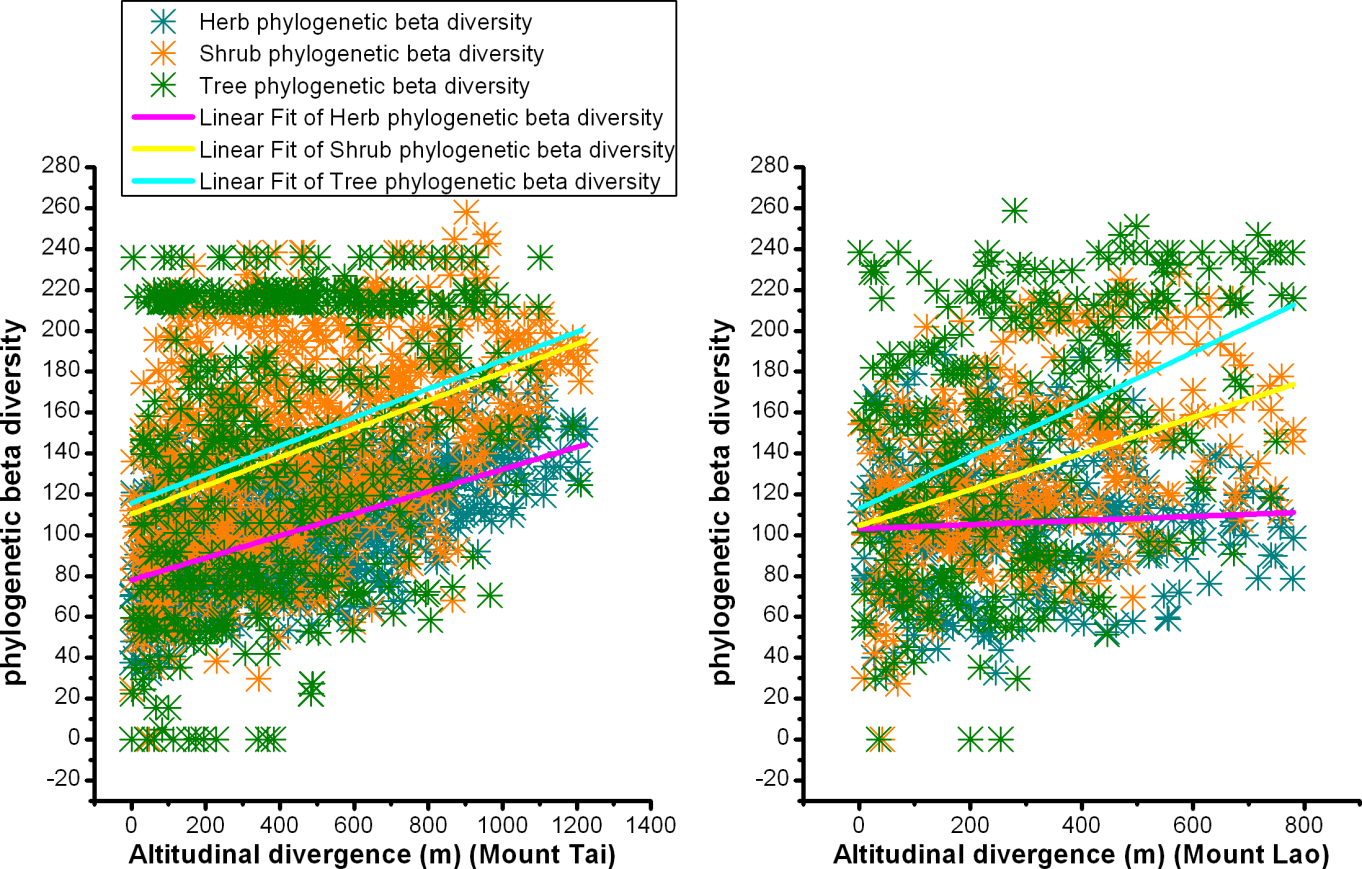
Relationship between phylogenetic distance and altitudinal divergence for trees, shrubs, and herbs on Mount Tai and Mount Lao, China.

The phylogenetic distance consistently increased with increased altitudinal divergence in our study, although this trend was not significant for herbs on Mount Lao. On Mount Tai, the regression slope between phylogenetic distances and altitudinal divergence was 0.06975, 0.06938, and 0.05419 for trees, shrubs, and herbs, respectively. On Mount Lao, the regression slope between phylogenetic distances and altitudinal divergence was 0.12758, 0.08851, and 0.01016 for trees, shrubs and herbs, respectively (Fig 4).

### Variation in phylogenetic community structure along elevation gradients

**Fig 5:**
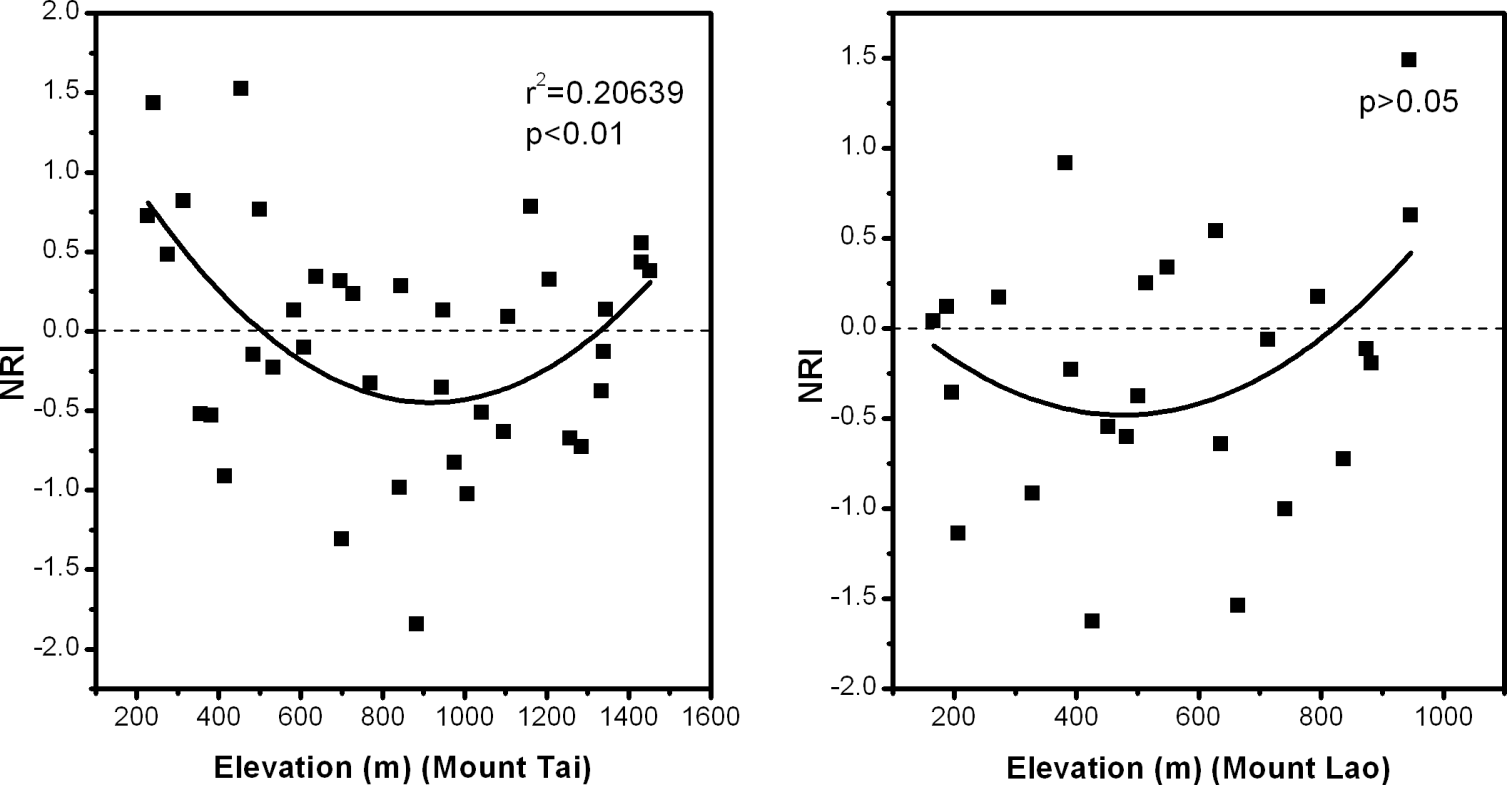
The changes in phylogenetic community structure (NRI) along elevation gradientson Mount Tai and Mount Lao, China.

The phylogenetic structure of plant communities exhibited an inverted hump-shaped pattern on Mount Tai. However, no clear pattern was detected from low to high elevation on Mount Lao (Fig 5).

## Discussion

The patterns of total species richness in response to elevation gradientson Mount Tai and Mount Lao are consistent, with a monotonically decreasing pattern. However, the species richness for the plant groups presented have different altitudinal patterns (Fig 1). In this study, species richness of trees had unimodal altitudinal patterns across the elevation gradients in the two mountain systems. Climate is a conspicuous factor regulating species distribution and richness in many areas [35]. McCain [36] put forward a climatic model and proposed that hump-shaped diversity patterns might be attributed to optimal climate conditions (e.g. precipitation and temperature) at the middle elevations. High-altitude areas have lower temperature, which limits plant growth, while low-altitude areas lack adequate rainfall and cannot meet moisture requirements for plant growth. An optimal range of temperature and precipitation occurred in the middle elevations of our study sites. Favorable climatic conditions provide the most available primary productivity in the mountain ecosystem and support the survival of more species [37, 38]. In addition, the harsh climate conditions, e.g., strong wind, intense solar radiation, and low fertility soil, may prevent the appearance of some species at high altitude, while interspecific competition may eliminate other species and drive down biological diversity in mild climate conditions at low altitude [6].

Altitudinal patterns in shrubs richness and herbs richness varied. Three patterns were found in our research: a monotonically decreasing pattern, an inverted hump-shaped pattern, and no significant pattern (Fig 1). This finding may be due to shrubs and herbs in the understory layer being more sensitive to changes in the microenvironment, such as disturbance and canopy cover [39] (Table 1). Schmitt et al. [40] compared the plant species composition of two forest fragments in the mountainous highlands of the Great Rift Valley, and found that the two forests shared many of same woody plant species, but with obvious differences in herb layers. They proposed that herbs were affected not only by changes in moisture caused by the elevation gradient, but also by local changes in moisture caused by other factors such as insolation or small streams [41]. Mount Tai and Mount Lao are important nature reserves and tourist scenic spots. Tourist activities are the major disturbance sources and low elevations suffer more anthropogenic impacts on Mount Tai and Mount Lao. The areas with moderate impacts may have greater environmental heterogeneity, which offers species greater opportunities for establishment and growth [10]. Tourist activities such as camping, bushwalking and plant picking, are more likely to influence plant diversity in the understory of mountain forests. The trampling of tourists will reduce the cover or vigor of resident species, which can lead to an increase in resource availability in the understory [42]. More available resources could provide more ecological niches for colonization by new species. In addition, forest canopy density could influence light intensity, moisture, and temperature in the understory [43]. Forest communities with higher basal areas tend to have higher stand density and lack sufficient sunshine in the understory [44], which lead to lower species diversity. Therefore, the current diversity pattern of shrubs and herbs along the elevation gradient may be shaped by the interaction of altitude, anthropogenic disturbances, and canopy density.

The similarity of species composition decreased with increasing geographical distances for all plant groups in this study. The rate of species turnover with altitudinal divergence is faster for trees than for shrubs and herbs in the two mountain systems (Fig 3). Species with greater dispersal were less susceptible to the limitations of dispersal distance; they could colonize more broadly and have lower species turnover rates [16, 21]. Shrubs and herbs in the understory layer were easily disturbed by human activities. Human activities provide new mechanisms for the spread of understory plants through seed dispersal, increasing the probability of understory plants expanding their current distribution. Comparing the rate of species turnover between pteridophytes and spermatophytes, Hong [21] found that pteridophytes, whose propagules are spores dispersed by wind, have a lower distance-decay rate than spermatophytes, which are dispersed by seeds or fruits and are less vagile than pteridophytes. These results show that dispersal limitation plays an important role in structuring the spatial distribution of plants.

It is generally believed that species diversity is a good surrogate for phylogenetic diversity [45, 46]. Bryant (2008) explored taxon richness and phylogenetic diversity of soil bacteria and plants along an elevation gradient, and found that the patterns of taxon richness reflected those of phylogenetic diversity for both soil bacteria and plants [47]. However, in our study, the altitudinal patterns of species richness do not completely mirror phylogenetic diversity patterns. There is no monotonically decreasing trend for total phylogenetic diversity from low to high elevations (Figs 1 and 2). Besides contemporary climate, the controlling factors for phylogenetic diversity may include geological history. In the future, phylogenetic diversity pattern studies combining with environmental data and geological history should be conducted. In addition, conservation strategies based simply on taxon richness are not sufficient. Increasing plant communities’ phylogenetic diversity can enhance their ability to cope with climate change, through an increase in genetic diversity [48]. The selection of conservation areas should consider preserving high taxon richness, while maximizing phylogenetic diversity to improve diversification potential in the future [49]. Phylogenetic beta diversity and species beta diversity had a consistent altitudinal pattern (Figs 3 and 4). Phylogenetic beta diversity demonstrates how phylogenetic relatedness varies across environmental and geographical gradients. Phylogenetic relatedness declined as altitudinal divergence increased in our research. Phylogenetic beta diversity and the decay rate of phylogenetic similarity were both lower in herbs, which indicated herbaceous plants have close phylogenetic relationships among plant communities, have wider niche breadth, and are more widely distributed than woody plants.

Phylogenetic structure can reveal the prevailing ecological processes influencing the observed patterns of species diversity. Based on the assumption of phylogenetic niche conservatism, environmental filtering leads to coexisting species having similar physiological tolerances, resulting in phylogenetic clustering. Interspecific competition leads to a community with distantly related species, resulting in phylogenetic overdispersion [23, 50]. Qian et al. (2014) analyzed phylogenetic structure of angiosperm assemblages along elevation gradients in Changbaishan, China, found that angiosperm assemblages tended to become more phylogenetically clustered at higher elevations [51]. Graham et al. (2009), Pellissier et al. (2013) and Machac et al. (2011) studied phylogenetic structure of hummingbird communities, butterfly communities and ant communities, also found that communities were phylogenetically clustered at higher elevations [52–54]. In contrast, tree communities were found to have a tendency to be more phylogenetically overdispersed as elevation increased in Malesian mountain forests [25]. In our study, the phylogenetic structure of plant communities on Mount Lao did not display significant patterns along the elevation gradient, but plant communities on Mount Tai exhibited an inverted hump-shaped pattern (Fig 5). This result indicates that environmental filtering is the main driver of plant community assembly at high and low elevations on Mount Tai. High-altitude areas usually have lower temperature, while low-altitude areas usually lack adequate moisture. After filtering by harsh climate conditions, species surviving in high and low elevation areas are from few lineages that have evolved the ability to tolerance these habitats. Favorable climatic conditions usually occurred in the middle elevation areas and support the survival of more species. Therefore, inter-specific competition may be the main driver of plant community assembly in the middle elevations. Mount Lao is surrounded by the sea on two sides. The weather is tempered by the ocean, and this region has milder climate than Mount Tai. Therefore, the role of environmental filtering was not significant at both ends of the elevation gradient on Mount Lao. The emergence of the different phylogenetic structure patterns above is probably caused by differences in sampling scale, spatial distribution, taxonomic groups, or local climate conditions among different studies [8, 55]. Therefore, research into the phylogenetic structure patterns are insufficient and should be expanded to include different taxa and different regions in the future. In addition, a deeper understanding of the mechanisms driving current diversity patterns can be achieved, when sufficient environmental and functional traits data are available for these ecosystems.

## Conclusions

In this study, we identified the prevailing forces structuring the species distribution of plants across an elevation gradient on two mountains by using phylogenetic and traditional diversity analyses in combination. Phylogenetic structure analyses revealed that environmental filtering is the main driver of plant community assembly at high and low elevations on Mount Tai. However, the role of environmental filtering was not significant at both ends of the elevation gradient on Mount Lao where the climate is milder. At low elevation, anthropogenic disturbances contributed to the increase of plant diversity, especially for shrubs and herbs in understory layers, which are more sensitive to changes in microenvironment. Species beta diversity and phylogenetic beta diversity consistently increased as altitudinal divergence increased. However, the altitudinal patterns of species diversity did not completely mirror phylogenetic diversity patterns. Therefore, conservation strategies simply based on taxon richness are insufficient. The selection of protected areas should consider preserving high phylogenetic diversity to improve diversification potential in the face of global climate change.

## Supporting Information

S1 Fig Angiosperm phylogenetic tree for Mount Tai. (JPG)

S2 Fig Angiosperm phylogenetic tree for Mount Lao. (JPG)

S1 Table Geographic information for each of the 63 plots. (DOCX)

S2 Table Species list for each of the 63 plots. (DOCX)
