# Supplementary figures and images for: Altitudinal Patterns of Species Diversity and Phylogenetic Diversity across Temperate Mountain Forests of Northern China
Source: PLoS One. 2016 Jul 25;11(7):e0159995. doi: 10.1371/journal.pone.0159995 (PMC4959731; doi:10.1371/journal.pone.0159995)

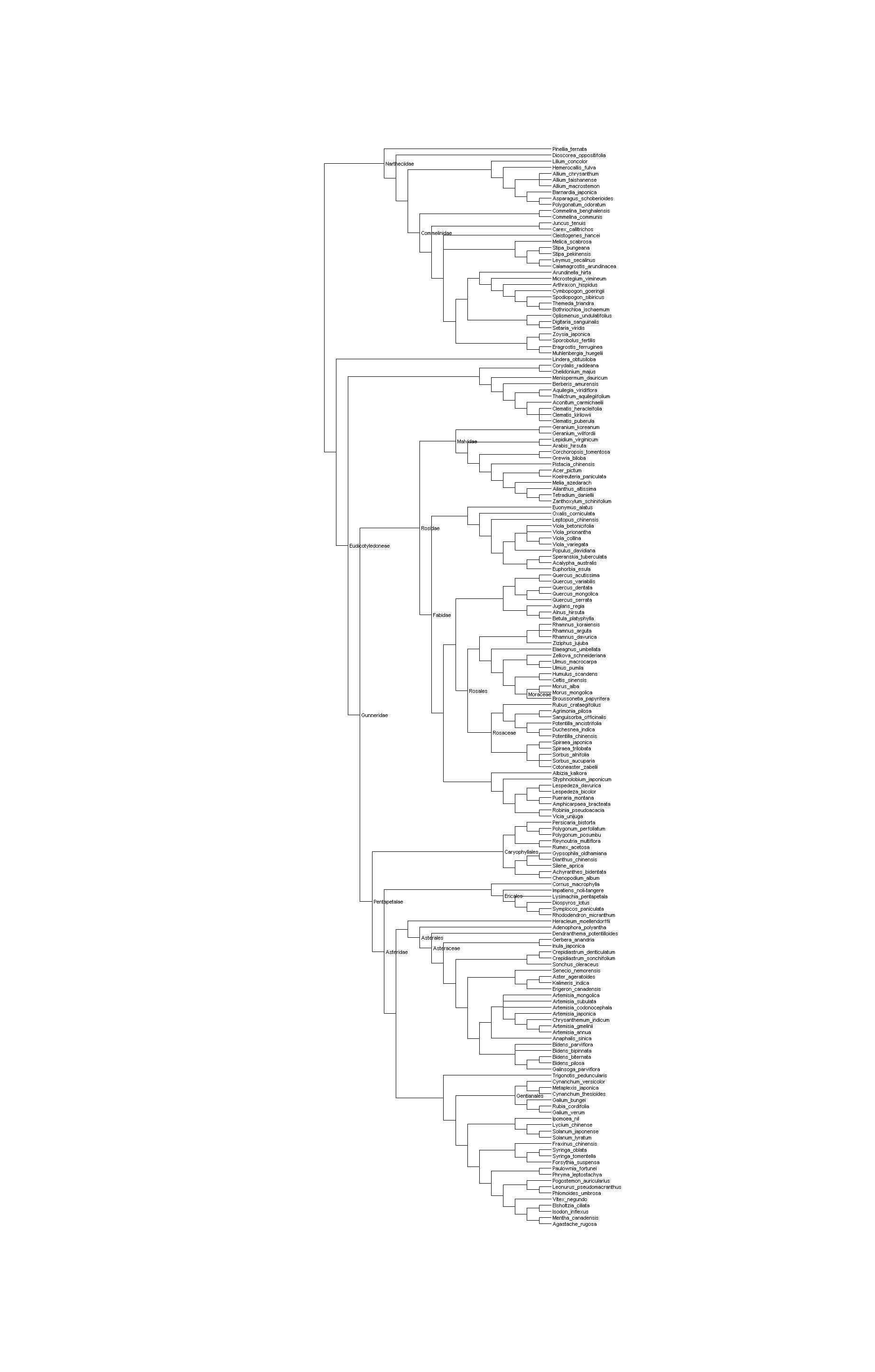

Supplement: S1 Fig — (JPG) [file pone.0159995.s001.jpg]

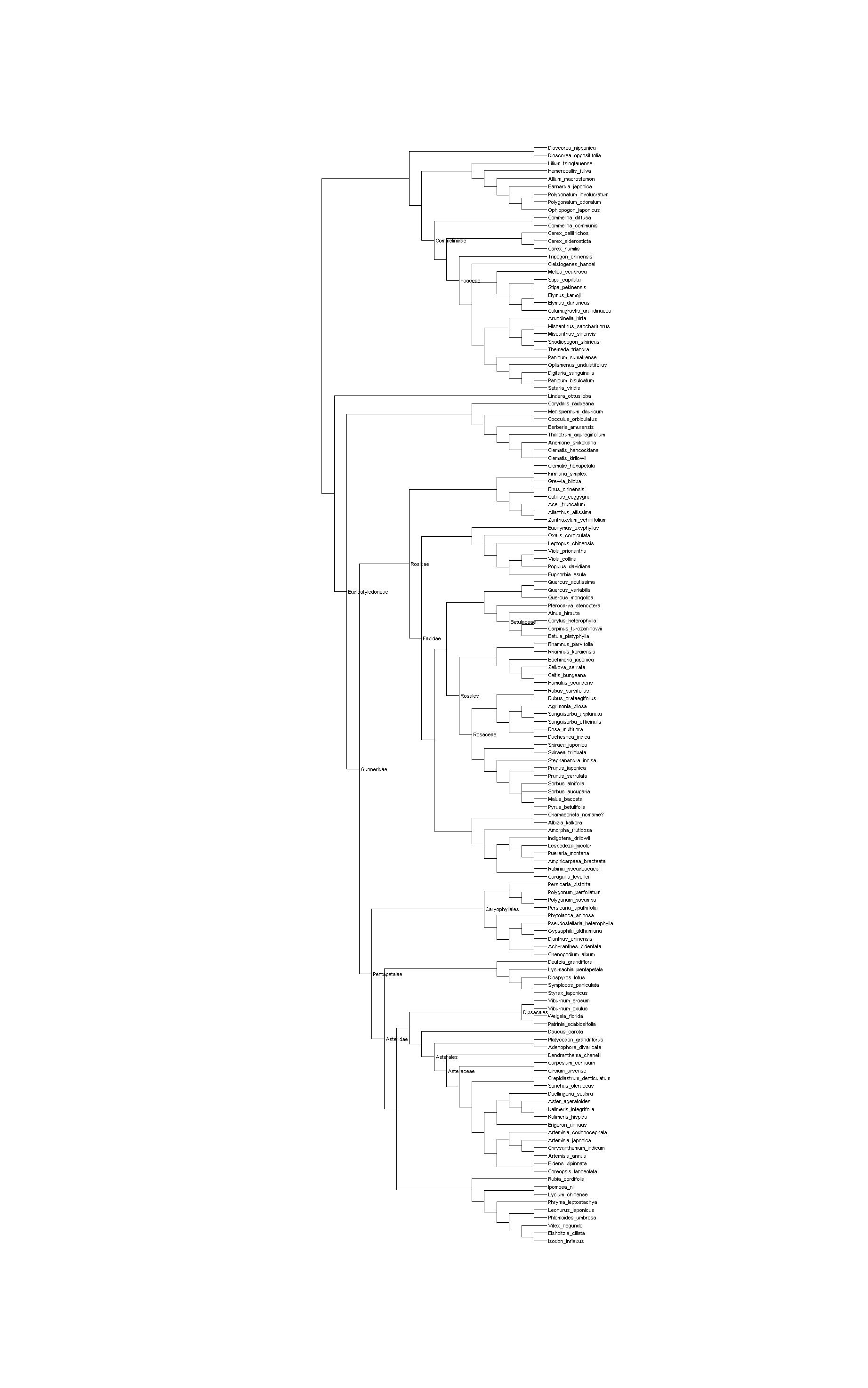

Supplement: S2 Fig — (JPG) [file pone.0159995.s002.jpg]
