# Supplementary material for: Altitudinal Patterns of Species Diversity and Phylogenetic Diversity across Temperate Mountain Forests of Northern China
Source: PLoS One. 2016 Jul 25;11(7):e0159995. doi: 10.1371/journal.pone.0159995 (PMC4959731; doi:10.1371/journal.pone.0159995)
Supplement: S1 Table — (DOCX) [file pone.0159995.s003.docx]

| Plot  number | Longitude  (°E) | Latitude  (°N) | Elevation  (m) | Aspect | Slope  (°) | Total basal area (m^2^) | Disturbance |
| --- | --- | --- | --- | --- | --- | --- | --- |
| 1 | 120.57917 | 36.08422 | 166 | 8 | 7 | 3331.10 | 3 |
| 2 | 120.60215 | 36.13753 | 188 | 7 | 12 | 3356.88 | 4 |
| 3 | 120.55432 | 36.18533 | 197 | 4 | 15 | 2805.44 | 3 |
| 4 | 120.55920 | 36.18632 | 207 | 4 | 3 | 3156.37 | 3 |
| 5 | 120.60438 | 36.14122 | 274 | 7 | 8 | 4555.16 | 3 |
| 6 | 120.61065 | 36.14870 | 328 | 6 | 8 | 2260.49 | 2 |
| 7 | 120.57282 | 36.14203 | 382 | 3 | 6 | 3805.44 | 3 |
| 8 | 120.57940 | 36.14538 | 391 | 3 | 6 | 2812.24 | 4 |
| 9 | 120.61653 | 36.15393 | 427 | 6 | 4 | 4250.24 | 2 |
| 10 | 120.57890 | 36.20155 | 452 | 8 | 10 | 3490.55 | 1 |
| 11 | 120.57883 | 36.20165 | 482 | 8 | 12 | 5884.56 | 1 |
| 12 | 120.62338 | 36.20187 | 501 | 4 | 15 | 2477.84 | 2 |
| 13 | 120.61707 | 36.20323 | 514 | 3 | 7 | 3274.34 | 3 |
| 14 | 120.62362 | 36.20397 | 549 | 8 | 4 | 2687.06 | 2 |
| 15 | 120.60870 | 36.20978 | 628 | 7 | 3 | 4704.84 | 2 |
| 16 | 120.56730 | 36.20213 | 636 | 1 | 18 | 13152.85 | 1 |
| 17 | 120.61723 | 36.20328 | 665 | 5 | 13 | 9312.52 | 1 |
| 18 | 120.59385 | 36.18055 | 714 | 5 | 8 | 4861.63 | 2 |
| 19 | 120.60913 | 36.19713 | 741 | 7 | 10 | 4043.24 | 2 |
| 20 | 120.61460 | 36.18600 | 795 | 7 | 8 | 7484.19 | 2 |
| 21 | 120.61727 | 36.18513 | 837 | 3 | 8 | 10018.00 | 2 |
| 22 | 120.61963 | 36.18465 | 874 | 3 | 15 | 7583.50 | 2 |
| 23 | 120.62430 | 36.17115 | 883 | 7 | 18 | 3717.91 | 2 |
| 24 | 120.61970 | 36.18223 | 945 | 1 | 6 | 6997.31 | 2 |
| 25 | 120.62535 | 36.17227 | 947 | 1 | 17 | 3739.35 | 2 |
| 26 | 117.11695 | 36.20787 | 228 | 8 | 5 | 5774.93 | 4 |
| 27 | 117.10452 | 36.20753 | 242 | 4 | 5 | 3811.86 | 4 |
| 28 | 117.12105 | 36.21100 | 276 | 8 | 4 | 5182.37 | 4 |
| 29 | 117.01774 | 36.26210 | 315 | 6 | 35 | 2077.00 | 3 |
| 30 | 117.13158 | 36.21583 | 356 | 1 | 2 | 2871.24 | 4 |
| 31 | 117.01611 | 36.25515 | 382 | 5 | 5 | 2386.94 | 2 |
| 32 | 117.01697 | 36.25322 | 415 | 8 | 5 | 5018.75 | 2 |
| 33 | 117.02732 | 36.27403 | 455 | 1 | 27 | 4730.25 | 3 |
| 34 | 117.05417 | 36.27778 | 485 | 6 | 25 | 3053.25 | 2 |
| 35 | 117.03270 | 36.27888 | 500 | 3 | 30 | 2876.72 | 2 |
| 36 | 117.03297 | 36.27463 | 534 | 6 | 24 | 2809.12 | 2 |
| 37 | 117.09530 | 36.23290 | 585 | 4 | 15 | 5316.59 | 3 |
| 38 | 117.09947 | 36.28442 | 609 | 8 | 4 | 1688.30 | 3 |
| 39 | 117.04322 | 36.26755 | 639 | 1 | 3 | 3284.55 | 3 |
| 40 | 117.10017 | 36.27990 | 698 | 8 | 35 | 1134.02 | 3 |
| 41 | 117.01327 | 36.26117 | 700 | 2 | 18 | 7017.20 | 4 |
| 42 | 117.10107 | 37.23498 | 728 | 4 | 15 | 5576.96 | 2 |
| 43 | 117.10451 | 36.23998 | 770 | 7 | 25 | 6267.24 | 2 |
| 44 | 117.10767 | 36.24097 | 841 | 1 | 30 | 6594.40 | 3 |
| 45 | 117.10730 | 36.24257 | 845 | 4 | 18 | 4742.67 | 2 |
| 46 | 117.07088 | 36.25103 | 883 | 7 | 27 | 4980.78 | 2 |
| 47 | 117.07070 | 36.24793 | 944 | 4 | 33 | 2954.47 | 3 |
| 48 | 117.07067 | 36.24745 | 948 | 4 | 19 | 4762.56 | 3 |
| 49 | 117.06998 | 36.24737 | 975 | 3 | 27 | 4569.19 | 1 |
| 50 | 117.08903 | 36.25773 | 1007 | 1 | 28 | 4806.33 | 2 |
| 51 | 117.08903 | 36.25814 | 1041 | 5 | 20 | 5352.12 | 3 |
| 52 | 117.08702 | 36.25877 | 1096 | 3 | 26 | 3713.81 | 4 |
| 53 | 117.08903 | 36.25884 | 1107 | 5 | 12 | 5832.74 | 2 |
| 54 | 117.08903 | 36.25955 | 1162 | 8 | 26 | 4969.77 | 1 |
| 55 | 117.09002 | 36.25944 | 1208 | 7 | 30 | 5495.40 | 3 |
| 56 | 117.09120 | 36.26025 | 1257 | 8 | 42 | 4926.02 | 1 |
| 57 | 117.09150 | 36.26176 | 1285 | 3 | 6 | 7182.78 | 3 |
| 58 | 117.09513 | 36.26225 | 1334 | 5 | 8 | 4946.00 | 2 |
| 59 | 117.09697 | 36.26118 | 1339 | 8 | 25 | 6105.07 | 1 |
| 60 | 117.09799 | 36.26054 | 1344 | 5 | 14 | 5912.78 | 2 |
| 61 | 117.09947 | 36.26165 | 1431 | 5 | 11 | 8720.44 | 1 |
| 62 | 117.09983 | 36.26158 | 1432 | 4 | 19 | 9985.96 | 3 |
| 63 | 117.10070 | 36.26200 | 1452 | 7 | 15 | 8924.83 | 1 |

Note：The aspect measurements were divided into 8 grades (1 = 337.6°– 22.5°, 2 = 22.6°– 67.5°, 3 = 292.6°– 337.5°, 4 = 67.6°– 112.5°, 5 = 247.6°–292.5°, 6 = 112.6°– 157.5°, 7 = 202.6°– 247.5°, 8 = 157.6°– 202.5°)
