# Supplementary material for: Altitudinal Patterns of Species Diversity and Phylogenetic Diversity across Temperate Mountain Forests of Northern China
Source: PLoS One. 2016 Jul 25;11(7):e0159995. doi: 10.1371/journal.pone.0159995 (PMC4959731; doi:10.1371/journal.pone.0159995)
Supplement: S2 Table — (DOCX) [file pone.0159995.s004.docx]

| Plot number | Species (Mount Lao) | Plot number | Species (Mount Tai) |
| --- | --- | --- | --- |
| 1 | Pinus thunbergii | 26 | Platycladus orientalis |
| 1 | Firmiana simplex | 26 | Vitex negundo |
| 1 | Populus davidiana | 26 | Grewia biloba |
| 1 | Lespedeza bicolor | 26 | Broussonetia papyrifera |
| 1 | Rhus chinensis | 26 | Zanthoxylum schinifolium |
| 1 | Zanthoxylum schinifolium | 26 | Ziziphus jujuba |
| 1 | Vitex negundo | 26 | Ailanthus altissima |
| 1 | Indigofera kirilowii | 26 | Lespedeza bicolor |
| 1 | Albizia kalkora | 26 | Oplismenus undulatifolius |
| 1 | Spodiopogon sibiricus | 26 | Solanum lyratum |
| 1 | Barnardia japonica | 26 | Lysimachia pentapetala |
| 1 | Gypsophila oldhamiana | 26 | Commelina benghalensis |
| 1 | Arundinella hirta | 26 | Viola prionantha |
| 1 | Allium macrostemon | 26 | Crepidiastrum denticulatum |
| 1 | Chrysanthemum indicum | 26 | Selaginella sinensis |
| 1 | Selaginella sinensis | 26 | Cleistogenes hancei |
| 1 | Calamagrostis arundinacea | 26 | Viola variegata |
| 1 | Miscanthus sinensis | 26 | Rubia cordifolia |
| 1 | Carex callitrichos | 26 | Arthraxon hispidus |
| 1 | Sanguisorba officinalis | 26 | Oxalis corniculata |
| 1 | Isodon inflexus | 26 | Ipomoea nil |
| 1 | Cleistogenes hancei | 26 | Setaria viridis |
| 1 | Lysimachia pentapetala | 26 | Bidens bipinnata |
| 1 | Sonchus oleraceus | 26 | Acalypha australis |
| 1 | Setaria viridis | 26 | Galinsoga parviflora |
| 1 | Bidens bipinnata | 26 | Artemisia annua |
| 1 | Erigeron annuus | 27 | Quercus acutissima |
| 1 | Coreopsis lanceolata | 27 | Platycladus orientalis |
| 1 | Chenopodium album | 27 | Quercus variabilis |
| 2 | Pinus thunbergii | 27 | Broussonetia papyrifera |
| 2 | Albizia kalkora | 27 | Vitex negundo |
| 2 | Lespedeza bicolor | 27 | Lespedeza bicolor |
| 2 | Vitex negundo | 27 | Ulmus pumila |
| 2 | Rubus parvifolius | 27 | Zanthoxylum schinifolium |
| 2 | Patrinia scabiosifolia | 27 | Ailanthus altissima |
| 2 | Lysimachia pentapetala | 27 | Grewia biloba |
| 2 | Gypsophila oldhamiana | 27 | Koelreuteria paniculata |
| 2 | Crepidiastrum denticulatum | 27 | Albizia kalkora |
| 2 | Cleistogenes hancei | 27 | Achyranthes bidentata |
| 2 | Selaginella sinensis | 27 | Rubia cordifolia |
| 2 | Chrysanthemum indicum | 27 | Crepidiastrum denticulatum |
| 2 | Spodiopogon sibiricus | 27 | Silene aprica |
| 2 | Miscanthus sinensis | 27 | Chrysanthemum indicum |
| 2 | Clematis hexapetala | 27 | Arthraxon hispidus |
| 2 | Allium macrostemon | 27 | Commelina benghalensis |
| 2 | Kalimeris hispida | 27 | Lysimachia pentapetala |
| 2 | Chamaecrista nomame | 27 | Chenopodium album |
| 2 | Digitaria sanguinalis | 27 | Ipomoea nil |
| 2 | Coreopsis lanceolata | 27 | Bidens bipinnata |
| 2 | Setaria viridis | 27 | Setaria viridis |
| 2 | Euphorbia esula | 28 | Platycladus orientalis |
| 3 | Robinia pseudoacacia | 28 | Broussonetia papyrifera |
| 3 | Quercus acutissima | 28 | Vitex negundo |
| 3 | Pinus thunbergii | 28 | Grewia biloba |
| 3 | Albizia kalkora | 28 | Lespedeza bicolor |
| 3 | Zanthoxylum schinifolium | 28 | Zanthoxylum schinifolium |
| 3 | Rhamnus koraiensis | 28 | Ziziphus jujuba |
| 3 | Vitex negundo | 28 | Ailanthus altissima |
| 3 | Lespedeza bicolor | 28 | Crepidiastrum denticulatum |
| 3 | Spiraea trilobata | 28 | Lysimachia pentapetala |
| 3 | Crepidiastrum denticulatum | 28 | Commelina benghalensis |
| 3 | Panicum bisulcatum | 28 | Crepidiastrum sonchifolium |
| 3 | Calamagrostis arundinacea | 28 | Solanum lyratum |
| 3 | Tripogon chinensis | 28 | Corchoropsis tomentosa |
| 3 | Dioscorea oppositifolia | 28 | Rubia cordifolia |
| 3 | Carex callitrichos | 28 | Chrysanthemum indicum |
| 3 | Kalimeris integrifolia | 28 | Carex callitrichos |
| 3 | Selaginella sinensis | 28 | Cleistogenes hancei |
| 3 | Cleistogenes hancei | 28 | Selaginella sinensis |
| 3 | Carpesium cernuum | 28 | Viola prionantha |
| 3 | Spodiopogon sibiricus | 28 | Gypsophila oldhamiana |
| 3 | Lysimachia pentapetala | 28 | Arthraxon hispidus |
| 3 | Artemisia codonocephala | 28 | Achyranthes bidentata |
| 3 | Commelina communis | 28 | Artemisia annua |
| 3 | Themeda triandra | 28 | Chenopodium album |
| 3 | Bidens bipinnata | 28 | Oxalis corniculata |
| 4 | Robinia pseudoacacia | 28 | Setaria viridis |
| 4 | Acer truncatum | 28 | Acalypha australis |
| 4 | Quercus acutissima | 28 | Bidens bipinnata |
| 4 | Zanthoxylum schinifolium | 28 | Digitaria sanguinalis |
| 4 | Rubus crataegifolius | 29 | Pinus densiflora |
| 4 | Grewia biloba | 29 | Vitex negundo |
| 4 | Carex callitrichos | 29 | Ziziphus jujuba |
| 4 | Dioscorea oppositifolia | 29 | Lespedeza bicolor |
| 4 | Viola prionantha | 29 | Koelreuteria paniculata |
| 4 | Duchesnea indica | 29 | Ulmus pumila |
| 4 | Crepidiastrum denticulatum | 29 | Albizia kalkora |
| 4 | Thalictrum aquilegiifolium | 29 | Potentilla chinensis |
| 4 | Commelina communis | 29 | Artemisia gmelinii |
| 4 | Leonurus japonicus | 29 | Selaginella sinensis |
| 4 | Boehmeria japonica | 29 | Crepidiastrum sonchifolium |
| 4 | Calamagrostis arundinacea | 29 | Arthraxon hispidus |
| 4 | Tripogon chinensis | 29 | Spodiopogon sibiricus |
| 4 | Achyranthes bidentata | 29 | Dianthus chinensis |
| 4 | Cleistogenes hancei | 29 | Cynanchum thesioides |
| 4 | Oplismenus undulatifolius | 29 | Crepidiastrum denticulatum |
| 4 | Oxalis corniculata | 29 | Artemisia japonica |
| 4 | Phytolacca acinosa | 29 | Cleistogenes hancei |
| 4 | Ipomoea nil | 29 | Bidens bipinnata |
| 5 | Alnus hirsuta | 29 | Themeda triandra |
| 5 | Pinus thunbergii | 29 | Oxalis corniculata |
| 5 | Pterocarya stenoptera | 30 | Quercus acutissima |
| 5 | Pyrus betulifolia | 30 | Acer pictum |
| 5 | Albizia kalkora | 30 | Grewia biloba |
| 5 | Quercus variabilis | 30 | Vitex negundo |
| 5 | Vitex negundo | 30 | Broussonetia papyrifera |
| 5 | Grewia biloba | 30 | Quercus variabilis |
| 5 | Rhus chinensis | 30 | Albizia kalkora |
| 5 | Rubus parvifolius | 30 | Commelina communis |
| 5 | Amorpha fruticosa | 30 | Arthraxon hispidus |
| 5 | Spodiopogon sibiricus | 30 | Lysimachia pentapetala |
| 5 | Artemisia codonocephala | 30 | Chrysanthemum indicum |
| 5 | Rubia cordifolia | 30 | Bidens parviflora |
| 5 | Thalictrum aquilegiifolium | 30 | Achyranthes bidentata |
| 5 | Clematis kirilowii | 30 | Trigonotis peduncularis |
| 5 | Isodon inflexus | 30 | Juncus tenuis |
| 5 | Boehmeria japonica | 30 | Rubia cordifolia |
| 5 | Phryma leptostachya | 30 | Commelina benghalensis |
| 5 | Dioscorea oppositifolia | 30 | Cynanchum thesioides |
| 5 | Corydalis raddeana | 30 | Bidens bipinnata |
| 5 | Achyranthes bidentata | 30 | Oxalis corniculata |
| 5 | Pueraria montana | 30 | Chenopodium album |
| 5 | Commelina communis | 30 | Setaria viridis |
| 5 | Cleistogenes hancei | 30 | Digitaria sanguinalis |
| 5 | Aster ageratoides | 30 | Acalypha australis |
| 5 | Menispermum dauricum | 30 | Themeda triandra |
| 5 | Calamagrostis arundinacea | 31 | Pinus densiflora |
| 5 | Humulus scandens | 31 | Vitex negundo |
| 5 | Oxalis corniculata | 31 | Bothriochloa ischaemum |
| 6 | Pinus thunbergii | 31 | Barnardia japonica |
| 6 | Rhus chinensis | 31 | Arthraxon hispidus |
| 6 | Populus davidiana | 31 | Pinellia ternata |
| 6 | Alnus hirsuta | 31 | Cynanchum versicolor |
| 6 | Lespedeza bicolor | 31 | Selaginella sinensis |
| 6 | Prunus japonica | 31 | Crepidiastrum denticulatum |
| 6 | Indigofera kirilowii | 31 | Artemisia codonocephala |
| 6 | Diospyros lotus | 31 | Artemisia gmelinii |
| 6 | Prunus serrulata | 31 | Agrimonia pilosa |
| 6 | Spodiopogon sibiricus | 31 | Dioscorea oppositifolia |
| 6 | Sanguisorba applanata | 31 | Sporobolus fertilis |
| 6 | Clematis hexapetala | 31 | Zoysia japonica |
| 6 | Pueraria montana | 31 | Setaria viridis |
| 6 | Cleistogenes hancei | 31 | Bidens biternata |
| 6 | Carex callitrichos | 31 | Oxalis corniculata |
| 6 | Selaginella sinensis | 31 | Digitaria sanguinalis |
| 6 | Crepidiastrum denticulatum | 31 | Erigeron canadensis |
| 6 | Dioscorea oppositifolia | 32 | Quercus acutissima |
| 6 | Chrysanthemum indicum | 32 | Pinus densiflora |
| 6 | Commelina communis | 32 | Quercus variabilis |
| 6 | Rubia cordifolia | 32 | Vitex negundo |
| 6 | Hemerocallis fulva | 32 | Grewia biloba |
| 7 | Alnus hirsuta | 32 | Crepidiastrum denticulatum |
| 7 | Pinus densiflora | 32 | Oplismenus undulatifolius |
| 7 | Ailanthus altissima | 32 | Lysimachia pentapetala |
| 7 | Lespedeza bicolor | 32 | Chrysanthemum indicum |
| 7 | Zanthoxylum schinifolium | 32 | Crepidiastrum sonchifolium |
| 7 | Rosa multiflora | 32 | Allium macrostemon |
| 7 | Albizia kalkora | 32 | Adenophora polyantha |
| 7 | Amorpha fruticosa | 32 | Viola betonicifolia |
| 7 | Patrinia scabiosifolia | 32 | Carex callitrichos |
| 7 | Sanguisorba officinalis | 32 | Pinellia ternata |
| 7 | Isodon inflexus | 32 | Setaria viridis |
| 7 | Spodiopogon sibiricus | 33 | Quercus acutissima |
| 7 | Miscanthus sinensis | 33 | Morus mongolica |
| 7 | Cocculus orbiculatus | 33 | Vitex negundo |
| 7 | Cirsium arvense | 33 | Ulmus macrocarpa |
| 7 | Selaginella sinensis | 33 | Robinia pseudoacacia |
| 8 | Alnus hirsuta | 33 | Albizia kalkora |
| 8 | Pinus thunbergii | 33 | Crepidiastrum denticulatum |
| 8 | Rhus chinensis | 33 | Carex callitrichos |
| 8 | Lespedeza bicolor | 33 | Gypsophila oldhamiana |
| 8 | Zanthoxylum schinifolium | 33 | Cynanchum versicolor |
| 8 | Rosa multiflora | 33 | Rubia cordifolia |
| 8 | Spiraea japonica | 33 | Pogostemon auricularius |
| 8 | Rubus crataegifolius | 33 | Dendranthema potentilloides |
| 8 | Amorpha fruticosa | 33 | Leonurus pseudomacranthus |
| 8 | Artemisia japonica | 33 | Oplismenus undulatifolius |
| 8 | Sanguisorba officinalis | 33 | Oxalis corniculata |
| 8 | Isodon inflexus | 33 | Bidens pilosa |
| 8 | Spodiopogon sibiricus | 34 | Pinus tabuliformis |
| 8 | Carex callitrichos | 34 | Quercus variabilis |
| 8 | Miscanthus sacchariflorus | 34 | Ailanthus altissima |
| 8 | Barnardia japonica | 34 | Pinus thunbergii |
| 8 | Carex humilis | 34 | Morus mongolica |
| 8 | Kalimeris integrifolia | 34 | Pinus densiflora |
| 8 | Doellingeria scabra | 34 | Vitex negundo |
| 8 | Carex siderosticta | 34 | Lespedeza bicolor |
| 8 | Thalictrum aquilegiifolium | 34 | Quercus acutissima |
| 8 | Elsholtzia ciliata | 34 | Leptopus chinensis |
| 8 | Daucus carota | 34 | Forsythia suspensa |
| 9 | Platycladus orientalis | 34 | Cleistogenes hancei |
| 9 | Rhus chinensis | 34 | Juncus tenuis |
| 9 | Alnus hirsuta | 34 | Cynanchum versicolor |
| 9 | Pinus thunbergii | 34 | Crepidiastrum denticulatum |
| 9 | Larix kaempferi | 34 | Artemisia gmelinii |
| 9 | Pinus densiflora | 34 | Reynoutria multiflora |
| 9 | Leptopus chinensis | 34 | Galium bungei |
| 9 | Rosa multiflora | 34 | Selaginella sinensis |
| 9 | Grewia biloba | 34 | Lysimachia pentapetala |
| 9 | Robinia pseudoacacia | 34 | Cynanchum thesioides |
| 9 | Oplismenus undulatifolius | 34 | Rubia cordifolia |
| 9 | Anisocampium niponicum | 34 | Oplismenus undulatifolius |
| 9 | Carex callitrichos | 34 | Cymbopogon goeringii |
| 9 | Dioscorea oppositifolia | 34 | Allium taishanense |
| 9 | Crepidiastrum denticulatum | 34 | Euphorbia esula |
| 9 | Ophiopogon japonicus | 34 | Themeda triandra |
| 9 | Commelina communis | 35 | Robinia pseudoacacia |
| 9 | Clematis kirilowii | 35 | Zanthoxylum schinifolium |
| 9 | Rubia cordifolia | 35 | Quercus variabilis |
| 9 | Spodiopogon sibiricus | 35 | Vitex negundo |
| 9 | Oxalis corniculata | 35 | Grewia biloba |
| 9 | Coreopsis lanceolata | 35 | Spiraea japonica |
| 10 | Quercus acutissima | 35 | Ziziphus jujuba |
| 10 | Robinia pseudoacacia | 35 | Albizia kalkora |
| 10 | Pinus densiflora | 35 | Melica scabrosa |
| 10 | Albizia kalkora | 35 | Oplismenus undulatifolius |
| 10 | Lespedeza bicolor | 35 | Rubia cordifolia |
| 10 | Rosa multiflora | 35 | Achyranthes bidentata |
| 10 | Zanthoxylum schinifolium | 35 | Amphicarpaea bracteata |
| 10 | Amorpha fruticosa | 35 | Sonchus oleraceus |
| 10 | Stipa capillata | 36 | Platycladus orientalis |
| 10 | Elymus dahuricus | 36 | Pinus tabuliformis |
| 10 | Gypsophila oldhamiana | 36 | Styphnolobium japonicum |
| 10 | Thalictrum aquilegiifolium | 36 | Vitex negundo |
| 10 | Viola prionantha | 36 | Spiraea trilobata |
| 10 | Pseudostellaria heterophylla | 36 | Lespedeza davurica |
| 10 | Cleistogenes hancei | 36 | Ailanthus altissima |
| 10 | Melica scabrosa | 36 | Anaphalis sinica |
| 10 | Dianthus chinensis | 36 | Carex callitrichos |
| 10 | Rubia cordifolia | 36 | Leymus secalinus |
| 10 | Ophiopogon japonicus | 36 | Lysimachia pentapetala |
| 10 | Hemerocallis fulva | 36 | Arthraxon hispidus |
| 11 | Albizia kalkora | 36 | Crepidiastrum denticulatum |
| 11 | Quercus acutissima | 36 | Gypsophila oldhamiana |
| 11 | Robinia pseudoacacia | 36 | Rubia cordifolia |
| 11 | Diospyros lotus | 36 | Dendranthema potentilloides |
| 11 | Lespedeza bicolor | 36 | Allium chrysanthum |
| 11 | Zanthoxylum schinifolium | 36 | Cleistogenes hancei |
| 11 | Grewia biloba | 36 | Bidens parviflora |
| 11 | Rhamnus parvifolia | 36 | Artemisia gmelinii |
| 11 | Rosa multiflora | 36 | Allium taishanense |
| 11 | Polygonum posumbu | 36 | Stipa pekinensis |
| 11 | Cleistogenes hancei | 36 | Oplismenus undulatifolius |
| 11 | Dioscorea nipponica | 36 | Artemisia subulata |
| 11 | Melica scabrosa | 36 | Dianthus chinensis |
| 11 | Commelina communis | 36 | Inula japonica |
| 11 | Polygonum perfoliatum | 36 | Spodiopogon sibiricus |
| 11 | Pseudostellaria heterophylla | 36 | Themeda triandra |
| 11 | Carex siderosticta | 36 | Digitaria sanguinalis |
| 11 | Anisocampium niponicum | 37 | Robinia pseudoacacia |
| 11 | Persicaria lapathifolia | 37 | Platycladus orientalis |
| 11 | Elymus dahuricus | 37 | Populus davidiana |
| 12 | Sorbus alnifolia | 37 | Quercus acutissima |
| 12 | Styrax japonicus | 37 | Vitex negundo |
| 12 | Rhus chinensis | 37 | Grewia biloba |
| 12 | Robinia pseudoacacia | 37 | Ailanthus altissima |
| 12 | Quercus acutissima | 37 | Quercus variabilis |
| 12 | Larix kaempferi | 37 | Ulmus pumila |
| 12 | Pinus tabuliformis | 37 | Morus alba |
| 12 | Pinus densiflora | 37 | Broussonetia papyrifera |
| 12 | Lespedeza bicolor | 37 | Quercus dentata |
| 12 | Caragana leveillei | 37 | Achyranthes bidentata |
| 12 | Stephanandra incisa | 37 | Oplismenus undulatifolius |
| 12 | Symplocos paniculata | 37 | Chrysanthemum indicum |
| 12 | Rhamnus koraiensis | 37 | Cleistogenes hancei |
| 12 | Lindera obtusiloba | 37 | Commelina communis |
| 12 | Zanthoxylum schinifolium | 37 | Carex callitrichos |
| 12 | Prunus japonica | 37 | Artemisia codonocephala |
| 12 | Oplismenus undulatifolius | 37 | Amphicarpaea bracteata |
| 12 | Clematis kirilowii | 37 | Corydalis raddeana |
| 12 | Sanguisorba officinalis | 37 | Dioscorea oppositifolia |
| 12 | Isodon inflexus | 37 | Geranium wilfordii |
| 12 | Platycodon grandiflorus | 37 | Viola prionantha |
| 12 | Spodiopogon sibiricus | 37 | Oxalis corniculata |
| 12 | Carex callitrichos | 37 | Humulus scandens |
| 12 | Elymus kamoji | 37 | Bidens bipinnata |
| 12 | Stipa pekinensis | 37 | Chenopodium album |
| 12 | Thalictrum aquilegiifolium | 38 | Quercus acutissima |
| 13 | Pinus tabuliformis | 38 | Pinus armandii |
| 13 | Pinus densiflora | 38 | Vitex negundo |
| 13 | Alnus hirsuta | 38 | Forsythia suspensa |
| 13 | Lespedeza bicolor | 38 | Spiraea japonica |
| 13 | Rhamnus koraiensis | 38 | Lespedeza bicolor |
| 13 | Zanthoxylum schinifolium | 38 | Fraxinus chinensis |
| 13 | Rosa multiflora | 38 | Rubus crataegifolius |
| 13 | Rubus crataegifolius | 38 | Oplismenus undulatifolius |
| 13 | Albizia kalkora | 38 | Carex callitrichos |
| 13 | Robinia pseudoacacia | 38 | Isodon inflexus |
| 13 | Melica scabrosa | 38 | Bidens parviflora |
| 13 | Kalimeris integrifolia | 38 | Stipa pekinensis |
| 13 | Stipa capillata | 38 | Phryma leptostachya |
| 13 | Sanguisorba officinalis | 38 | Viola collina |
| 13 | Adenophora divaricata | 38 | Arundinella hirta |
| 13 | Cleistogenes hancei | 38 | Sanguisorba officinalis |
| 13 | Rubia cordifolia | 38 | Viola prionantha |
| 13 | Thalictrum aquilegiifolium | 38 | Allium macrostemon |
| 13 | Ophiopogon japonicus | 38 | Thalictrum aquilegiifolium |
| 13 | Dendranthema chanetii | 38 | Speranskia tuberculata |
| 13 | Elymus dahuricus | 38 | Anaphalis sinica |
| 13 | Artemisia annua | 38 | Selaginella sinensis |
| 13 | Humulus scandens | 38 | Cleistogenes hancei |
| 14 | Styrax japonicus | 38 | Artemisia annua |
| 14 | Sorbus alnifolia | 38 | Bidens bipinnata |
| 14 | Alnus hirsuta | 39 | Robinia pseudoacacia |
| 14 | Quercus acutissima | 39 | Pinus densiflora |
| 14 | Pinus thunbergii | 39 | Juglans regia |
| 14 | Quercus mongolica | 39 | Broussonetia papyrifera |
| 14 | Larix kaempferi | 39 | Vitex negundo |
| 14 | Robinia pseudoacacia | 39 | Grewia biloba |
| 14 | Pinus tabuliformis | 39 | Spiraea japonica |
| 14 | Pinus densiflora | 39 | Fraxinus chinensis |
| 14 | Caragana leveillei | 39 | Ziziphus jujuba |
| 14 | Celtis bungeana | 39 | Rhamnus arguta |
| 14 | Stephanandra incisa | 39 | Artemisia codonocephala |
| 14 | Lindera obtusiloba | 39 | Clematis puberula |
| 14 | Zanthoxylum schinifolium | 39 | Oplismenus undulatifolius |
| 14 | Symplocos paniculata | 39 | Rubia cordifolia |
| 14 | Rosa multiflora | 39 | Melica scabrosa |
| 14 | Isodon inflexus | 39 | Amphicarpaea bracteata |
| 14 | Carex callitrichos | 39 | Geranium koreanum |
| 14 | Calamagrostis arundinacea | 39 | Carex callitrichos |
| 14 | Sanguisorba officinalis | 39 | Anisocampium niponicum |
| 14 | Oplismenus undulatifolius | 39 | Lilium concolor |
| 14 | Rubia cordifolia | 39 | Clematis kirilowii |
| 14 | Thalictrum aquilegiifolium | 39 | Polygonum perfoliatum |
| 15 | Pinus tabuliformis | 39 | Crepidiastrum sonchifolium |
| 15 | Pinus densiflora | 39 | Pogostemon auricularius |
| 15 | Larix kaempferi | 39 | Humulus scandens |
| 15 | Rhus chinensis | 39 | Ipomoea nil |
| 15 | Alnus hirsuta | 39 | Chenopodium album |
| 15 | Robinia pseudoacacia | 39 | Lepidium virginicum |
| 15 | Quercus acutissima | 40 | Pinus tabuliformis |
| 15 | Symplocos paniculata | 40 | Spiraea japonica |
| 15 | Quercus mongolica | 40 | Lespedeza bicolor |
| 15 | Caragana leveillei | 40 | Forsythia suspensa |
| 15 | Euonymus oxyphyllus | 40 | Euonymus alatus |
| 15 | Rhamnus parvifolia | 40 | Rubus crataegifolius |
| 15 | Sorbus aucuparia | 40 | Leptopus chinensis |
| 15 | Deutzia grandiflora | 40 | Vitex negundo |
| 15 | Zanthoxylum schinifolium | 40 | Carex callitrichos |
| 15 | Stephanandra incisa | 40 | Spodiopogon sibiricus |
| 15 | Albizia kalkora | 40 | Arundinella hirta |
| 15 | Oplismenus undulatifolius | 40 | Sanguisorba officinalis |
| 15 | Sanguisorba officinalis | 40 | Isodon inflexus |
| 15 | Carex callitrichos | 40 | Crepidiastrum denticulatum |
| 15 | Thalictrum aquilegiifolium | 40 | Crepidiastrum sonchifolium |
| 15 | Allium macrostemon | 40 | Selaginella sinensis |
| 15 | Spodiopogon sibiricus | 40 | Oplismenus undulatifolius |
| 15 | Sonchus oleraceus | 40 | Achyranthes bidentata |
| 16 | Chamaecyparis pisifera | 40 | Chrysanthemum indicum |
| 16 | Larix kaempferi | 40 | Anaphalis sinica |
| 16 | Lindera obtusiloba | 40 | Anisocampium niponicum |
| 16 | Carpinus turczaninowii | 40 | Arthraxon hispidus |
| 16 | Diospyros lotus | 40 | Eragrostis ferruginea |
| 16 | Betula platyphylla | 40 | Viola prionantha |
| 16 | Grewia biloba | 40 | Stipa pekinensis |
| 16 | Corylus heterophylla | 40 | Kalimeris indica |
| 16 | Spiraea japonica | 40 | Setaria viridis |
| 16 | Prunus serrulata | 40 | Bidens bipinnata |
| 16 | Malus baccata | 40 | Digitaria sanguinalis |
| 16 | Oplismenus undulatifolius | 41 | Robinia pseudoacacia |
| 16 | Lilium tsingtauense | 41 | Pinus tabuliformis |
| 16 | Polygonatum odoratum | 41 | Juglans regia |
| 16 | Crepidiastrum denticulatum | 41 | Grewia biloba |
| 17 | Chamaecyparis pisifera | 41 | Rubus crataegifolius |
| 17 | Quercus acutissima | 41 | Spiraea japonica |
| 17 | Malus baccata | 41 | Oplismenus undulatifolius |
| 17 | Lindera obtusiloba | 41 | Melica scabrosa |
| 17 | Spiraea japonica | 41 | Aquilegia viridiflora |
| 17 | Grewia biloba | 41 | Polygonatum odoratum |
| 17 | Zanthoxylum schinifolium | 41 | Viola prionantha |
| 17 | Oplismenus undulatifolius | 41 | Crepidiastrum denticulatum |
| 17 | Rubia cordifolia | 41 | Clematis puberula |
| 17 | Viola prionantha | 41 | Carex callitrichos |
| 17 | Commelina diffusa | 41 | Chrysanthemum indicum |
| 17 | Stipa capillata | 41 | Duchesnea indica |
| 17 | Carex siderosticta | 41 | Ipomoea nil |
| 18 | Larix kaempferi | 41 | Hemerocallis fulva |
| 18 | Pinus densiflora | 41 | Chenopodium album |
| 18 | Alnus hirsuta | 42 | Platycladus orientalis |
| 18 | Lespedeza bicolor | 42 | Pinus tabuliformis |
| 18 | Spiraea japonica | 42 | Robinia pseudoacacia |
| 18 | Rubus crataegifolius | 42 | Grewia biloba |
| 18 | Symplocos paniculata | 42 | Broussonetia papyrifera |
| 18 | Robinia pseudoacacia | 42 | Melia azedarach |
| 18 | Miscanthus sinensis | 42 | Morus alba |
| 18 | Panicum sumatrense | 42 | Vitex negundo |
| 18 | Agrimonia pilosa | 42 | Celtis sinensis |
| 18 | Isodon inflexus | 42 | Morus mongolica |
| 18 | Platycodon grandiflorus | 42 | Achyranthes bidentata |
| 18 | Thalictrum aquilegiifolium | 42 | Crepidiastrum denticulatum |
| 18 | Viola collina | 42 | Oplismenus undulatifolius |
| 18 | Sanguisorba officinalis | 42 | Calamagrostis arundinacea |
| 18 | Doellingeria scabra | 42 | Spodiopogon sibiricus |
| 18 | Allium macrostemon | 42 | Artemisia codonocephala |
| 18 | Calamagrostis arundinacea | 42 | Rubia cordifolia |
| 19 | Larix kaempferi | 42 | Phryma leptostachya |
| 19 | Symplocos paniculata | 42 | Pinellia ternata |
| 19 | Zelkova serrata | 42 | Bidens bipinnata |
| 19 | Pinus thunbergii | 42 | Chenopodium album |
| 19 | Lindera obtusiloba | 43 | Robinia pseudoacacia |
| 19 | Sorbus alnifolia | 43 | Quercus acutissima |
| 19 | Rhus chinensis | 43 | Diospyros lotus |
| 19 | Stephanandra incisa | 43 | Koelreuteria paniculata |
| 19 | Rhamnus koraiensis | 43 | Celtis sinensis |
| 19 | Deutzia grandiflora | 43 | Oplismenus undulatifolius |
| 19 | Euonymus oxyphyllus | 43 | Cleistogenes hancei |
| 19 | Prunus japonica | 43 | Thalictrum aquilegiifolium |
| 19 | Aster ageratoides | 43 | Achyranthes bidentata |
| 19 | Allium macrostemon | 43 | Arundinella hirta |
| 19 | Rubia cordifolia | 43 | Corydalis raddeana |
| 19 | Sanguisorba officinalis | 43 | Rubia cordifolia |
| 19 | Viola collina | 43 | Chelidonium majus |
| 19 | Carex callitrichos | 43 | Amphicarpaea bracteata |
| 19 | Oplismenus undulatifolius | 43 | Chrysanthemum indicum |
| 19 | Platycodon grandiflorus | 43 | Humulus scandens |
| 19 | Crepidiastrum denticulatum | 43 | Sonchus oleraceus |
| 19 | Miscanthus sinensis | 44 | Platycladus orientalis |
| 19 | Spodiopogon sibiricus | 44 | Robinia pseudoacacia |
| 19 | Lilium tsingtauense | 44 | Grewia biloba |
| 19 | Chrysanthemum indicum | 44 | Celtis sinensis |
| 19 | Thalictrum aquilegiifolium | 44 | Crepidiastrum denticulatum |
| 19 | Stipa pekinensis | 44 | Calamagrostis arundinacea |
| 19 | Ipomoea nil | 44 | Oplismenus undulatifolius |
| 20 | Larix kaempferi | 44 | Carex callitrichos |
| 20 | Sorbus alnifolia | 44 | Chelidonium majus |
| 20 | Symplocos paniculata | 44 | Amphicarpaea bracteata |
| 20 | Quercus acutissima | 44 | Corydalis raddeana |
| 20 | Viburnum opulus | 44 | Artemisia codonocephala |
| 20 | Spiraea japonica | 44 | Melica scabrosa |
| 20 | Stephanandra incisa | 44 | Thalictrum aquilegiifolium |
| 20 | Persicaria bistorta | 44 | Rubia cordifolia |
| 20 | Sanguisorba officinalis | 44 | Chenopodium album |
| 20 | Agrimonia pilosa | 44 | Euphorbia esula |
| 20 | Amphicarpaea bracteata | 45 | Robinia pseudoacacia |
| 20 | Rubia cordifolia | 45 | Diospyros lotus |
| 20 | Carex callitrichos | 45 | Lespedeza bicolor |
| 20 | Thalictrum aquilegiifolium | 45 | Euonymus alatus |
| 20 | Anisocampium niponicum | 45 | Celtis sinensis |
| 20 | Aster ageratoides | 45 | Calamagrostis arundinacea |
| 20 | Isodon inflexus | 45 | Menispermum dauricum |
| 20 | Duchesnea indica | 45 | Chelidonium majus |
| 20 | Stipa pekinensis | 45 | Oplismenus undulatifolius |
| 20 | Calamagrostis arundinacea | 45 | Amphicarpaea bracteata |
| 20 | Melica scabrosa | 45 | Achyranthes bidentata |
| 20 | Panicum sumatrense | 45 | Arabis hirsuta |
| 21 | Larix kaempferi | 45 | Crepidiastrum denticulatum |
| 21 | Stephanandra incisa | 45 | Rubia cordifolia |
| 21 | Symplocos paniculata | 45 | Corydalis raddeana |
| 21 | Spiraea japonica | 45 | Dioscorea oppositifolia |
| 21 | Viburnum opulus | 45 | Artemisia codonocephala |
| 21 | Phlomoides umbrosa | 45 | Pueraria montana |
| 21 | Carex callitrichos | 45 | Rumex acetosa |
| 21 | Panicum sumatrense | 45 | Bidens bipinnata |
| 21 | Amphicarpaea bracteata | 45 | Mentha canadensis |
| 21 | Polygonatum involucratum | 45 | Humulus scandens |
| 21 | Daucus carota | 46 | Pinus tabuliformis |
| 22 | Larix kaempferi | 46 | Pinus koraiensis |
| 22 | Sorbus alnifolia | 46 | Larix principis-rupprechtii |
| 22 | Stephanandra incisa | 46 | Pinus armandii |
| 22 | Rosa multiflora | 46 | Robinia pseudoacacia |
| 22 | Zanthoxylum schinifolium | 46 | Ailanthus altissima |
| 22 | Viburnum opulus | 46 | Forsythia suspensa |
| 22 | Spiraea japonica | 46 | Spiraea trilobata |
| 22 | Adenophora divaricata | 46 | Syringa oblata |
| 22 | Panicum sumatrense | 46 | Euonymus alatus |
| 22 | Phlomoides umbrosa | 46 | Oplismenus undulatifolius |
| 22 | Thalictrum aquilegiifolium | 46 | Corydalis raddeana |
| 22 | Carex callitrichos | 46 | Melica scabrosa |
| 22 | Anisocampium niponicum | 46 | Crepidiastrum denticulatum |
| 22 | Amphicarpaea bracteata | 46 | Pinellia ternata |
| 22 | Tripogon chinensis | 46 | Polygonatum odoratum |
| 23 | Quercus acutissima | 46 | Viola prionantha |
| 23 | Larix kaempferi | 46 | Carex callitrichos |
| 23 | Lindera obtusiloba | 46 | Metaplexis japonica |
| 23 | Sorbus alnifolia | 46 | Commelina communis |
| 23 | Symplocos paniculata | 46 | Muhlenbergia huegelii |
| 23 | Euonymus oxyphyllus | 46 | Amphicarpaea bracteata |
| 23 | Spiraea japonica | 47 | Betula platyphylla |
| 23 | Rubus crataegifolius | 47 | Pinus tabuliformis |
| 23 | Zanthoxylum schinifolium | 47 | Fraxinus chinensis |
| 23 | Rhamnus parvifolia | 47 | Syringa oblata |
| 23 | Caragana leveillei | 47 | Spiraea trilobata |
| 23 | Lespedeza bicolor | 47 | Rhamnus davurica |
| 23 | Rosa multiflora | 47 | Pinus armandii |
| 23 | Sanguisorba officinalis | 47 | Cotoneaster zabelii |
| 23 | Allium macrostemon | 47 | Forsythia suspensa |
| 23 | Carex callitrichos | 47 | Pistacia chinensis |
| 23 | Panicum sumatrense | 47 | Lespedeza bicolor |
| 23 | Thalictrum aquilegiifolium | 47 | Quercus acutissima |
| 23 | Aster ageratoides | 47 | Oplismenus undulatifolius |
| 23 | Lilium tsingtauense | 47 | Kalimeris indica |
| 23 | Crepidiastrum denticulatum | 47 | Thalictrum aquilegiifolium |
| 23 | Selaginella sinensis | 47 | Viola collina |
| 23 | Phlomoides umbrosa | 47 | Sanguisorba officinalis |
| 23 | Agrimonia pilosa | 47 | Allium taishanense |
| 23 | Anemone shikokiana | 47 | Crepidiastrum denticulatum |
| 24 | Larix kaempferi | 47 | Melica scabrosa |
| 24 | Symplocos paniculata | 47 | Carex callitrichos |
| 24 | Spiraea japonica | 47 | Artemisia codonocephala |
| 24 | Lycium chinense | 47 | Rubia cordifolia |
| 24 | Viburnum opulus | 47 | Amphicarpaea bracteata |
| 24 | Lespedeza bicolor | 47 | Metaplexis japonica |
| 24 | Rosa multiflora | 47 | Lysimachia pentapetala |
| 24 | Aster ageratoides | 47 | Duchesnea indica |
| 24 | Amphicarpaea bracteata | 47 | Commelina communis |
| 24 | Carex callitrichos | 47 | Dendranthema potentilloides |
| 24 | Panicum sumatrense | 47 | Polygonatum odoratum |
| 24 | Anisocampium niponicum | 47 | Muhlenbergia huegelii |
| 24 | Persicaria bistorta | 47 | Elsholtzia ciliata |
| 24 | Isodon inflexus | 47 | Chenopodium album |
| 24 | Doellingeria scabra | 48 | Pinus tabuliformis |
| 24 | Daucus carota | 48 | Betula platyphylla |
| 24 | Erigeron annuus | 48 | Pinus armandii |
| 25 | Larix kaempferi | 48 | Quercus variabilis |
| 25 | Quercus acutissima | 48 | Fraxinus chinensis |
| 25 | Cotinus coggygria | 48 | Larix principis-rupprechtii |
| 25 | Sorbus alnifolia | 48 | Alnus hirsuta |
| 25 | Viburnum erosum | 48 | Spiraea trilobata |
| 25 | Stephanandra incisa | 48 | Elaeagnus umbellata |
| 25 | Euonymus oxyphyllus | 48 | Forsythia suspensa |
| 25 | Lindera obtusiloba | 48 | Muhlenbergia huegelii |
| 25 | Weigela florida | 48 | Commelina communis |
| 25 | Caragana leveillei | 48 | Oplismenus undulatifolius |
| 25 | Berberis amurensis | 48 | Aquilegia viridiflora |
| 25 | Zanthoxylum schinifolium | 48 | Bidens parviflora |
| 25 | Prunus japonica | 48 | Agrimonia pilosa |
| 25 | Panicum sumatrense | 48 | Crepidiastrum denticulatum |
| 25 | Clematis hancockiana | 48 | Corydalis raddeana |
| 25 | Aster ageratoides | 48 | Melica scabrosa |
| 25 | Carex callitrichos | 48 | Artemisia codonocephala |
| 25 | Viola collina | 48 | Duchesnea indica |
| 25 | Artemisia codonocephala | 48 | Rubia cordifolia |
| 25 | Sanguisorba officinalis | 48 | Microstegium vimineum |
| 25 | Anemone shikokiana | 48 | Viola collina |
| 25 | Phlomoides umbrosa | 48 | Isodon inflexus |
| 25 | Agrimonia pilosa | 48 | Lysimachia pentapetala |
| 25 | Thalictrum aquilegiifolium | 48 | Sanguisorba officinalis |
|  |  | 48 | Anisocampium niponicum |
|  |  | 48 | Chenopodium album |
|  |  | 48 | Elsholtzia ciliata |
|  |  | 48 | Galium verum |
|  |  | 48 | Setaria viridis |
|  |  | 48 | Humulus scandens |
|  |  | 49 | Alnus hirsuta |
|  |  | 49 | Pinus armandii |
|  |  | 49 | Zelkova schneideriana |
|  |  | 49 | Larix principis-rupprechtii |
|  |  | 49 | Forsythia suspensa |
|  |  | 49 | Spiraea trilobata |
|  |  | 49 | Corydalis raddeana |
|  |  | 49 | Microstegium vimineum |
|  |  | 49 | Oplismenus undulatifolius |
|  |  | 49 | Duchesnea indica |
|  |  | 49 | Commelina communis |
|  |  | 49 | Amphicarpaea bracteata |
|  |  | 49 | Viola collina |
|  |  | 49 | Anisocampium niponicum |
|  |  | 49 | Isodon inflexus |
|  |  | 49 | Polygonum posumbu |
|  |  | 49 | Crepidiastrum denticulatum |
|  |  | 49 | Pinellia ternata |
|  |  | 49 | Elsholtzia ciliata |
|  |  | 49 | Chenopodium album |
|  |  | 50 | Pinus tabuliformis |
|  |  | 50 | Robinia pseudoacacia |
|  |  | 50 | Larix principis-rupprechtii |
|  |  | 50 | Juglans regia |
|  |  | 50 | Sorbus aucuparia |
|  |  | 50 | Quercus mongolica |
|  |  | 50 | Rubus crataegifolius |
|  |  | 50 | Lycium chinense |
|  |  | 50 | Rhododendron micranthum |
|  |  | 50 | Arundinella hirta |
|  |  | 50 | Menispermum dauricum |
|  |  | 50 | Corydalis raddeana |
|  |  | 50 | Amphicarpaea bracteata |
|  |  | 50 | Anisocampium niponicum |
|  |  | 50 | Dioscorea oppositifolia |
|  |  | 50 | Calamagrostis arundinacea |
|  |  | 50 | Artemisia codonocephala |
|  |  | 50 | Carex callitrichos |
|  |  | 50 | Aster ageratoides |
|  |  | 50 | Thalictrum aquilegiifolium |
|  |  | 50 | Clematis heracleifolia |
|  |  | 50 | Solanum japonense |
|  |  | 51 | Larix principis-rupprechtii |
|  |  | 51 | Pinus tabuliformis |
|  |  | 51 | Robinia pseudoacacia |
|  |  | 51 | Alnus hirsuta |
|  |  | 51 | Sorbus aucuparia |
|  |  | 51 | Quercus mongolica |
|  |  | 51 | Pinus armandii |
|  |  | 51 | Rubus crataegifolius |
|  |  | 51 | Symplocos paniculata |
|  |  | 51 | Arundinella hirta |
|  |  | 51 | Anisocampium niponicum |
|  |  | 51 | Artemisia mongolica |
|  |  | 51 | Aster ageratoides |
|  |  | 51 | Dioscorea oppositifolia |
|  |  | 51 | Carex callitrichos |
|  |  | 51 | Menispermum dauricum |
|  |  | 51 | Corydalis raddeana |
|  |  | 51 | Amphicarpaea bracteata |
|  |  | 51 | Solanum japonense |
|  |  | 51 | Humulus scandens |
|  |  | 52 | Sorbus aucuparia |
|  |  | 52 | Pinus tabuliformis |
|  |  | 52 | Robinia pseudoacacia |
|  |  | 52 | Rubus crataegifolius |
|  |  | 52 | Aster ageratoides |
|  |  | 52 | Clematis kirilowii |
|  |  | 52 | Crepidiastrum denticulatum |
|  |  | 52 | Anisocampium niponicum |
|  |  | 52 | Isodon inflexus |
|  |  | 52 | Gerbera anandria |
|  |  | 52 | Corydalis raddeana |
|  |  | 52 | Amphicarpaea bracteata |
|  |  | 52 | Solanum lyratum |
|  |  | 52 | Arundinella hirta |
|  |  | 52 | Polygonatum odoratum |
|  |  | 52 | Melica scabrosa |
|  |  | 52 | Menispermum dauricum |
|  |  | 53 | Pinus tabuliformis |
|  |  | 53 | Robinia pseudoacacia |
|  |  | 53 | Pinus armandii |
|  |  | 53 | Larix principis-rupprechtii |
|  |  | 53 | Sorbus aucuparia |
|  |  | 53 | Quercus mongolica |
|  |  | 53 | Lespedeza bicolor |
|  |  | 53 | Quercus dentata |
|  |  | 53 | Rubus crataegifolius |
|  |  | 53 | Tetradium daniellii |
|  |  | 53 | Broussonetia papyrifera |
|  |  | 53 | Agastache rugosa |
|  |  | 53 | Corydalis raddeana |
|  |  | 53 | Arundinella hirta |
|  |  | 53 | Menispermum dauricum |
|  |  | 53 | Crepidiastrum denticulatum |
|  |  | 53 | Anisocampium niponicum |
|  |  | 53 | Amphicarpaea bracteata |
|  |  | 53 | Crepidiastrum sonchifolium |
|  |  | 53 | Stipa bungeana |
|  |  | 53 | Rubia cordifolia |
|  |  | 53 | Aster ageratoides |
|  |  | 53 | Solanum lyratum |
|  |  | 53 | Solanum japonense |
|  |  | 53 | Carex callitrichos |
|  |  | 53 | Hemerocallis fulva |
|  |  | 54 | Quercus mongolica |
|  |  | 54 | Pinus tabuliformis |
|  |  | 54 | Robinia pseudoacacia |
|  |  | 54 | Acer pictum |
|  |  | 54 | Paulownia fortunei |
|  |  | 54 | Rubus crataegifolius |
|  |  | 54 | Arundinella hirta |
|  |  | 54 | Crepidiastrum denticulatum |
|  |  | 54 | Amphicarpaea bracteata |
|  |  | 54 | Viola collina |
|  |  | 54 | Aster ageratoides |
|  |  | 54 | Corydalis raddeana |
|  |  | 54 | Artemisia mongolica |
|  |  | 54 | Isodon inflexus |
|  |  | 54 | Solanum japonense |
|  |  | 54 | Asparagus schoberioides |
|  |  | 54 | Senecio nemorensis |
|  |  | 55 | Sorbus alnifolia |
|  |  | 55 | Pinus tabuliformis |
|  |  | 55 | Quercus acutissima |
|  |  | 55 | Sorbus aucuparia |
|  |  | 55 | Quercus serrata |
|  |  | 55 | Forsythia suspensa |
|  |  | 55 | Rubus crataegifolius |
|  |  | 55 | Lespedeza bicolor |
|  |  | 55 | Rhamnus koraiensis |
|  |  | 55 | Euonymus alatus |
|  |  | 55 | Isodon inflexus |
|  |  | 55 | Arundinella hirta |
|  |  | 55 | Thalictrum aquilegiifolium |
|  |  | 55 | Carex callitrichos |
|  |  | 55 | Clematis heracleifolia |
|  |  | 55 | Calamagrostis arundinacea |
|  |  | 55 | Solanum japonense |
|  |  | 55 | Artemisia mongolica |
|  |  | 55 | Amphicarpaea bracteata |
|  |  | 55 | Sanguisorba officinalis |
|  |  | 56 | Pinus tabuliformis |
|  |  | 56 | Sorbus alnifolia |
|  |  | 56 | Forsythia suspensa |
|  |  | 56 | Rhododendron micranthum |
|  |  | 56 | Rubus crataegifolius |
|  |  | 56 | Lespedeza bicolor |
|  |  | 56 | Carex callitrichos |
|  |  | 56 | Aster ageratoides |
|  |  | 56 | Calamagrostis arundinacea |
|  |  | 56 | Persicaria bistorta |
|  |  | 56 | Artemisia mongolica |
|  |  | 56 | Aquilegia viridiflora |
|  |  | 56 | Viola collina |
|  |  | 56 | Isodon inflexus |
|  |  | 56 | Arundinella hirta |
|  |  | 56 | Corydalis raddeana |
|  |  | 56 | Sanguisorba officinalis |
|  |  | 56 | Clematis heracleifolia |
|  |  | 56 | Polygonatum odoratum |
|  |  | 56 | Solanum japonense |
|  |  | 56 | Senecio nemorensis |
|  |  | 57 | Larix principis-rupprechtii |
|  |  | 57 | Pinus tabuliformis |
|  |  | 57 | Rubus crataegifolius |
|  |  | 57 | Lespedeza bicolor |
|  |  | 57 | Lindera obtusiloba |
|  |  | 57 | Berberis amurensis |
|  |  | 57 | Corydalis raddeana |
|  |  | 57 | Arundinella hirta |
|  |  | 57 | Stipa bungeana |
|  |  | 57 | Aster ageratoides |
|  |  | 57 | Amphicarpaea bracteata |
|  |  | 57 | Geranium koreanum |
|  |  | 57 | Persicaria bistorta |
|  |  | 57 | Viola collina |
|  |  | 57 | Isodon inflexus |
|  |  | 57 | Potentilla ancistrifolia |
|  |  | 57 | Senecio nemorensis |
|  |  | 57 | Carex callitrichos |
|  |  | 57 | Agrimonia pilosa |
|  |  | 57 | Sanguisorba officinalis |
|  |  | 58 | Larix principis-rupprechtii |
|  |  | 58 | Ulmus macrocarpa |
|  |  | 58 | Berberis amurensis |
|  |  | 58 | Corydalis raddeana |
|  |  | 58 | Artemisia codonocephala |
|  |  | 58 | Geranium wilfordii |
|  |  | 58 | Amphicarpaea bracteata |
|  |  | 58 | Impatiens noli-tangere |
|  |  | 58 | Menispermum dauricum |
|  |  | 58 | Arundinella hirta |
|  |  | 58 | Persicaria bistorta |
|  |  | 59 | Pinus armandii |
|  |  | 59 | Sorbus alnifolia |
|  |  | 59 | Pinus tabuliformis |
|  |  | 59 | Sorbus aucuparia |
|  |  | 59 | Larix principis-rupprechtii |
|  |  | 59 | Symplocos paniculata |
|  |  | 59 | Berberis amurensis |
|  |  | 59 | Euonymus alatus |
|  |  | 59 | Cornus macrophylla |
|  |  | 59 | Lespedeza bicolor |
|  |  | 59 | Senecio nemorensis |
|  |  | 59 | Carex callitrichos |
|  |  | 59 | Thalictrum aquilegiifolium |
|  |  | 59 | Arundinella hirta |
|  |  | 59 | Corydalis raddeana |
|  |  | 59 | Artemisia mongolica |
|  |  | 59 | Calamagrostis arundinacea |
|  |  | 59 | Aster ageratoides |
|  |  | 59 | Vicia unijuga |
|  |  | 59 | Heracleum moellendorffii |
|  |  | 59 | Sanguisorba officinalis |
|  |  | 59 | Viola collina |
|  |  | 60 | Pinus armandii |
|  |  | 60 | Pinus tabuliformis |
|  |  | 60 | Pistacia chinensis |
|  |  | 60 | Fraxinus chinensis |
|  |  | 60 | Berberis amurensis |
|  |  | 60 | Rhamnus koraiensis |
|  |  | 60 | Rubus crataegifolius |
|  |  | 60 | Sorbus alnifolia |
|  |  | 60 | Amphicarpaea bracteata |
|  |  | 60 | Geranium koreanum |
|  |  | 60 | Artemisia mongolica |
|  |  | 60 | Thalictrum aquilegiifolium |
|  |  | 60 | Corydalis raddeana |
|  |  | 60 | Aconitum carmichaelii |
|  |  | 60 | Arundinella hirta |
|  |  | 60 | Clematis heracleifolia |
|  |  | 61 | Larix principis-rupprechtii |
|  |  | 61 | Alnus hirsuta |
|  |  | 61 | Pinus armandii |
|  |  | 61 | Fraxinus chinensis |
|  |  | 61 | Pinus tabuliformis |
|  |  | 61 | Spiraea japonica |
|  |  | 61 | Berberis amurensis |
|  |  | 61 | Syringa tomentella |
|  |  | 61 | Phlomoides umbrosa |
|  |  | 61 | Melica scabrosa |
|  |  | 61 | Corydalis raddeana |
|  |  | 61 | Viola collina |
|  |  | 61 | Aconitum carmichaelii |
|  |  | 61 | Kalimeris indica |
|  |  | 61 | Thalictrum aquilegiifolium |
|  |  | 61 | Impatiens noli-tangere |
|  |  | 61 | Vicia unijuga |
|  |  | 61 | Artemisia codonocephala |
|  |  | 62 | Alnus hirsuta |
|  |  | 62 | Pinus armandii |
|  |  | 62 | Larix principis-rupprechtii |
|  |  | 62 | Fraxinus chinensis |
|  |  | 62 | Syringa oblata |
|  |  | 62 | Spiraea japonica |
|  |  | 62 | Berberis amurensis |
|  |  | 62 | Syringa tomentella |
|  |  | 62 | Euonymus alatus |
|  |  | 62 | Phlomoides umbrosa |
|  |  | 62 | Corydalis raddeana |
|  |  | 62 | Amphicarpaea bracteata |
|  |  | 62 | Impatiens noli-tangere |
|  |  | 62 | Melica scabrosa |
|  |  | 62 | Agrimonia pilosa |
|  |  | 62 | Persicaria bistorta |
|  |  | 62 | Geranium wilfordii |
|  |  | 62 | Viola collina |
|  |  | 62 | Aconitum carmichaelii |
|  |  | 62 | Artemisia codonocephala |
|  |  | 62 | Kalimeris indica |
|  |  | 62 | Carex callitrichos |
|  |  | 62 | Thalictrum aquilegiifolium |
|  |  | 63 | Larix principis-rupprechtii |
|  |  | 63 | Alnus hirsuta |
|  |  | 63 | Euonymus alatus |
|  |  | 63 | Fraxinus chinensis |
|  |  | 63 | Spiraea japonica |
|  |  | 63 | Impatiens noli-tangere |
|  |  | 63 | Phlomoides umbrosa |
|  |  | 63 | Corydalis raddeana |
|  |  | 63 | Thalictrum aquilegiifolium |
|  |  | 63 | Menispermum dauricum |
|  |  | 63 | Melica scabrosa |
|  |  | 63 | Kalimeris indica |
|  |  | 63 | Amphicarpaea bracteata |
|  |  | 63 | Senecio nemorensis |
|  |  | 63 | Persicaria bistorta |
